# USP29-mediated HIF1α stabilization is associated with Sorafenib resistance of hepatocellular carcinoma cells by upregulating glycolysis

**DOI:** 10.1038/s41389-021-00338-7

**Published:** 2021-07-16

**Authors:** Ruize Gao, David Buechel, Ravi K. R. Kalathur, Marco F. Morini, Mairene Coto-Llerena, Caner Ercan, Salvatore Piscuoglio, Qian Chen, Tanja Blumer, Xueya Wang, Eva Dazert, Markus H. Heim, Michael N. Hall, Fengyuan Tang, Gerhard Christofori

**Affiliations:** 1 Department of Biomedicine, University of Basel, Basel, Switzerland; 2 Institute of Pathology, University Hospital Basel, Basel, Switzerland; 3 University Hospital Basel, Basel, Switzerland; 4 Biozentrum, University of Basel, Basel, Switzerland

**Keywords:** Liver cancer, Targeted therapies, Ubiquitylation

## Abstract

Understanding the mechanisms underlying evasive resistance in cancer is an unmet medical need to improve the efficacy of current therapies. In hepatocellular carcinoma (HCC), aberrant expression of hypoxia-inducible factor 1 α (HIF1α) and increased aerobic glycolysis metabolism are drivers of resistance to therapy with the multi-kinase inhibitor Sorafenib. However, it has remained unknown how HIF1α is activated and how its activity and the subsequent induction of aerobic glycolysis promote Sorafenib resistance in HCC. Here, we report the ubiquitin-specific peptidase USP29 as a new regulator of HIF1α and of aerobic glycolysis during the development of Sorafenib resistance in HCC. In particular, we identified USP29 as a critical deubiquitylase (DUB) of HIF1α, which directly deubiquitylates and stabilizes HIF1α and, thus, promotes its transcriptional activity. Among the transcriptional targets of HIF1α is the gene encoding hexokinase 2 (HK2), a key enzyme of the glycolytic pathway. The absence of USP29, and thus of HIF1α transcriptional activity, reduces the levels of aerobic glycolysis and restores sensitivity to Sorafenib in Sorafenib-resistant HCC cells in vitro and in xenograft transplantation mouse models in vivo. Notably, the absence of USP29 and high HK2 expression levels correlate with the response of HCC patients to Sorafenib therapy. Together, the data demonstrate that, as a DUB of HIF1α, USP29 promotes Sorafenib resistance in HCC cells, in parts by upregulating glycolysis, thereby opening new avenues for therapeutically targeting Sorafenib-resistant HCC in patients.

## Introduction

Liver cancer is the second leading cause of cancer-related death worldwide. Hepatocellular carcinoma (HCC) represents the most common type of primary malignant liver tumor, accounting for 90% of all liver cancers [1]. Unfortunately, only 30% of HCC patients are diagnosed at an early stage of carcinogenesis. Most of the patients are diagnosed at advanced stages, where surgical resection, allogeneic liver transplantation, or percutaneous tumor ablation are not applicable. Sorafenib, a multi-kinase inhibitor, is the standard of care treatment for advanced HCC patients, yet it prolongs the median overall survival and radiological progression by only ~3 months [2]. Comparable to many other targeted therapies, evasive resistance to Sorafenib is invariably observed in HCC patients. Therefore, a detailed understanding of how HCC cells respond to Sorafenib will not only help to improve the efficacy of Sorafenib therapy in HCC patients, but will also be critical to overcome the development of therapy resistance.

HIF1 (Hypoxia-inducible factor 1) is a well-known key regulator of cellular adaptive responses to hypoxia. Furthermore, it is a highly oncogenic transcription factor that promotes tumor growth via regulating global transcriptomic networks involved in tumor angiogenesis, metabolism, and therapy resistance [3]. Hypoxia and HIF1α play important roles in HCC development and relapse after chemotherapy [4]. The HIF1α protein is also found at high levels in tumors of HCC patients which are resistant to Sorafenib treatment [5]. As a heterodimeric transcription factor composed of HIF1α and HIF1β (ARNT), HIF’s transcriptional activity is regulated mainly at the level of HIF1α expression. The stability of HIF1α protein is regulated by the ubiquitin-proteasome system (UPS). Under normoxic conditions, HIF1α is hydroxylated by oxygen-dependent proline hydroxylases (PHDs), which earmarks it as a substrate of the ubiquitin E3 ligase von Hippel-Lindau (VHL). After sufficient ubiquitylation, it is degraded by the proteasome. Under hypoxic conditions, PHDs are not active, and HIF1α is not hydroxylated and ubiquitylated and, thus, stabilized to exert its transcriptional activities [6–10]. Recent studies have indicated that HIF1α can also be stabilized by deubiquitinating enzymes (DUBs) not only under hypoxic but also under normoxic conditions [6].

Aerobic glycolysis (aka Warburg effect) is a general metabolic feature of malignant tumors [11]. Aberrant glycolysis levels, including increased glucose uptake and lactate production, seem to be central for malignant progression of solid tumors, and HIF1α also has been implicated in the regulation of genes responsible for aberrant glycolysis [12]. Excessive glycolysis has also been reported to contribute to Sorafenib resistance in HCC cells [13,14,]. However, it has remained unclear how glycolysis is upregulated in Sorafenib-resistant HCC cells.

Ubiquitin-specific peptidase 29 (USP29) is a highly conserved DUB that belongs to the PH_USP37_like family. The PH_USP37_like family plays important role in cell proliferation and cancer growth. It consists of three members, USP26, USP29 and USP37, of which USP26 is a positive regulator of Androgen Receptor in prostate cancer cells [15], USP37 directly deubiquitinates and stabilizes c-Myc in lung cancer [16], and USP29 has been recognized as a regulator of the checkpoint adaptor Claspin [17]. However, the functional contribution of USP29 to tumorigenesis and therapy resistance has remained unexplored.

Here, we report the identification of USP29 as a regulator of HIF1α in HCC cells. Our data indicate that the USP29-HIF1α axis supports Sorafenib resistance by promoting glycolysis in HCC cells. The findings highlight USP29 and HIF1α as biomarkers for Sorafenib resistance in HCC and the USP29-HIF1α-glycolysis regulatory cascade as a potential therapeutic target to overcome Sorafenib resistance in HCC patients.

## Results

### Identification of HIF1α as a biomarker of Sorafenib-resistance

**Fig. 1:**
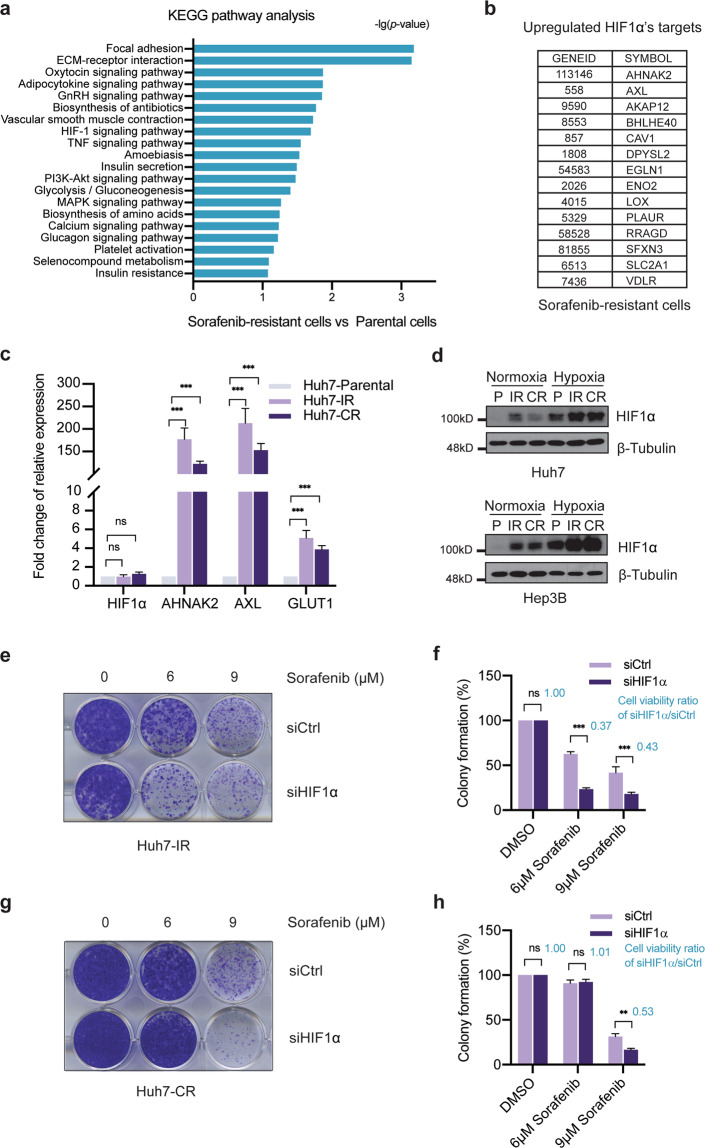
Identification of HIF1α as a biomarker of Sorafenib-resistance. **a** KEGG pathway analysis of the genes specifically upregulated in their expression in Sorafenib-resistant cells. The HIF-1 signaling pathway was identified as an upregulated pathway. **b** Top list of HIF1α target genes highly expressed in Sorafenib-resistant cells. **c** Huh7-IR and Huh7-CR cells expressed high mRNA levels of HIF1α target genes *HIF1α*, *AHNAK2*, *AXL*, and *GLUT1*. The transcripts of selected HIF1α target genes were quantified by quantitative RT-PCR. Fold increases are shown (*n* = 3 independent replicates). ns = not significant; *P < 0.05; **P < 0.01; ***P < 0.001; Student’s *t*-test. **d** Higher HIF1α protein levels were detected in Sorafenib-resistant Huh7-IR and Huh7-CR (upper panel) and Hep3B-IR and Hep3B-CR (lower panel) cells as compared to Huh7 and Hep3B parental (P) cells under either normoxic or hypoxic culture conditions. Immunoblotting for β-Tubulin was used as loading control. Results represent three independent replicative experiments. **e**–**h** Loss of HIF1α in Sorafenib-resistant Huh7-IR and Huh7-CR cells induced cell death upon Sorafenib treatment. Colony formation assays were performed with Huh7-IR (**e**) and Huh7-CR (**g**) cells transfected with either siCtrl or siHIF1α and treated with different concentrations of Sorafenib (0 μM, 6 μM, 9 μM) for 2 weeks. Colony formation was quantified by crystal violet staining (**f**, **h**). The ratio of cell viability between HIF1α-deficient and HIF1α-wildtype cells is given in blue numbers (**f**, **h**). n = 3 independent replicates. ns not significant; **P < 0.01; ***P < 0.001; Student’s *t*-test.

To uncover the molecular mechanisms underlying Sorafenib resistance in HCC, we first determined the IC50 values for Sorafenib in repressing the growth of patient-derived HCC cell lines (Suppl. Fig. 1a). We selected two of the most Sorafenib-susceptible cell lines (Huh7 and Hep3B) to establish cellular models of Sorafenib resistance by treating the cells with either increasing concentrations (IR) or a consistently high concentration (CR) of Sorafenib (Suppl.Fig. 1b) [18]. These treatments generated the Sorafenib-resistant cell lines Huh7-IR, Huh7-CR, Hep3B-IR, and Hep3B-CR with IC50 values of 10.7, 10.8, 7.2, and 8.3 μM, respectively, which are close to the clinically relevant Sorafenib concentration of 10 μM (Suppl. Fig. 1c).

Next, we performed whole transcriptome analysis of Sorafenib-responsive Huh7 and Hep3B parental cells and of the various Sorafenib-resistant cell lines, and determined genes differentially expressed between the parental cell lines and the Sorafenib-resistant cell lines (Suppl. Fig. 1d; Suppl. Table I). KEGG pathway analysis of the genes specifically expressed in Sorafenib-resistant cells identified hypoxia-inducible factor (HIF)-mediated signaling as a major pathway activated in Sorafenib-resistant cells (Fig. 1a). HIF1α is known to regulate a global adaptive transcriptional response to hypoxia and, as such, it is a critical oncoprotein in promoting tumor growth via regulating transcriptomic networks involved in angiogenesis, metabolism, and therapy resistance. To assess whether HIF1α and its transcriptional target genes contributed to Sorafenib resistance, we first identified the HIF1α target genes highly expressed in Sorafenib-resistant HCC cells (Fig. 1b). Indeed, quantitative RT-PCR analysis validated the high expression of a selection of prototype HIF1α target genes in Sorafenib-resistant Huh7-IR and CR cells as compared to Huh7-parental cells (Fig. 1c). Consistent with the increased expression of its target genes, HIF1α was found increased at the protein level in the Sorafenib-resistant cells as compared to their parental Huh7 and Hep3B cells, notably even under normoxic culture conditions (Fig. 1d).

To assess the functional role of HIF1α in driving Sorafenib resistance, we performed colony formation assays with Huh7-IR and Huh7-CR cells with and without siRNA-mediated depletion of HIF1α expression and in the presence of different concentrations of Sorafenib (Fig. 1e–h; Suppl. Fig. 2a, b). In line with a previous study showing a critical role of HIF1α in Sorafenib-naive cells [5], the results demonstrated that HIF1α was critically required for the maintenance of Sorafenib resistance in patient-derived HCC cell lines. HIF2α is another family member of HIFs known to promote tumorigenesis. However, siRNA-mediated depletion of HIF2α in both Huh7-IR and Huh7-CR cells had no impact on their resistance to Sorafenib treatment (Suppl. Fig. 2c–h). Together, these results suggest that HIF1α, but not HIF2α, sustains the resistance of HCC cells to Sorafenib therapy.

### USP29 stabilizes HIF1α

Next, we sought to examine how the activity of HIF1α is regulated in Sorafenib-resistant HCC cells. HIF1α’s activity is regulated predominantly at the post-transcriptional level, in particular by the UPS. In normoxia, the E3 ligase and tumor suppressor gene Von Hippel Lindau (VHL) ubiquitylates HIF1α and thereby marks it for proteasomal degradation. On the other hand, deubiquitylating enzymes (DUBs), such as USP8, USP28, and UCHL1, stabilize HIF1α under normoxia. Given that the HIF1α mRNA levels were not changed in the Sorafenib-resistant HCC cells, yet HIF1α protein levels were increased, we hypothesized that the HIF1α protein was stabilized via its level of ubiquitination.

**Fig. 2:**
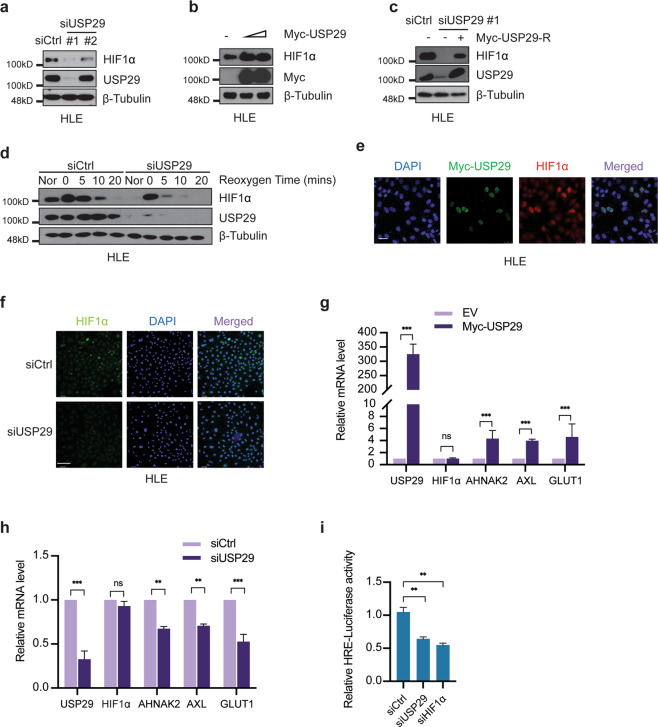
USP29 stabilizes HIF1α and promotes HIF1α’s transcriptional activity. **a** Depletion of USP29 diminished HIF1α protein levels in Sorafenib-resistant HLE cells. Two different siRNAs against USP29 (siUSP29#1 and siUSP29#2) were transfected into cells for the depletion of USP29. siUSP29#1 had a superior knockdown efficiency than siUSP29#2. Based on its high knockdown efficiency, siUSP29#1 was used for further experiments. Immunoblotting for β-Tubulin was used as loading control. Results represent three independent experiments. **b** USP29 promotes HIF1α protein stability. Myc-tagged USP29 was transfected into HLE cells, and endogenous HIF1α protein level was measured by immunoblotting. Immunoblotting for β-Tubulin was used as loading control. Results represent three independent replicative experiments. **c** Expression of an RNAi-resistant USP29 (Myc-USP29-R) rescued USP29 deficiency-induced instability of HIF1α. HLE cells were first transfected with siUSP29#1 and 24 h later with Myc-USP29-R or Empty-Vector. USP29 and HIF1α protein levels were determined by immunoblotting. Immunoblotting for β-Tubulin was used as loading control. Results represent three independent replicative experiments. **d** USP29 deficiency induces HIF1α protein degradation. HLE cells transfected with siCtrl or ON-TARGET siUSP29 were incubated in a hypoxia chamber (1% O_2_, 94% N_2_, 5% CO_2_) for 6 h and then moved to normoxia for 0, 5, 10, and 20 min. Culture in normoxia (Nor) was used as a control. Immunoblotting was used to visualize the kinetics of HIF1α degradation. Immunoblotting for USP29 was used to validate the knockdown efficiency and β-Tubulin as loading control. Results represent three independent replicative experiments. **e** USP29 promotes HIF1α stability and nuclear localization. A plasmid encoding for Myc-tagged USP29 was transfected into HLE cells and Myc-tagged USP29 and endogenous HIF1α were visualized by immunofluorescence microscopy analysis. Staining with DAPI was used to visualize nuclei. Results represent three independent experiments. Scale bar, 50 µm. **f** Loss of USP29 expression reduces HIF1α stability and nuclear localization. HLE cells were transfected with siCtrl or ON-TARGET siUSP29, and HIF1α was visualized by immunofluorescence microscopy for staining of endogenous HIF1α. DAPI staining was used to visualize nuclei. Scale bar, 132.5 µm. **g** USP29 promotes HIF1α transcriptional activity. Expression of the HIF1α target genes *AHNAK2, AXL, GLUT1* was examined in HLE cells transfected with a plasmid encoding for Myc-tagged USP29, and mRNA levels were determined by quantitative RT-PCR. Relative mRNA expression is shown. *n* = 3 independent replicates. ns = not significant; ***P < 0.001; Student’s *t*-test. **h** USP29 deficiency reduces HIF1α transcriptional activity. HLE cells were transfected with siCtrl or ON-TARGET siUSP29, and the expression of a panel of HIF1α transcriptional target genes was analyzed by quantitative RT-PCR. Relative mRNA expression is shown. *n* = 3 independent replicates. ns not significant; **P < 0.01; ***P < 0.001; Student’s *t*-test. **i** Loss of USP29 expression reduces HIF1α transcriptional activity. HLE cells were transfected with siCtrl, ON-TARGET siUSP29, and siHIF1α and 24 h later with plasmids carrying a HIF responsive element (HRE) driving the expression of *Firefly* luciferase (pGL4.42) and CMV promoter-driven *Renilla* luciferase (pRL-CMV) in a 10:1 mass ratio. Relative luciferase activities were measured by a dual-luciferase reporter assay. Results represent three independent experiments. **P < 0.01; Student’s *t*-test.

To test this hypothesis, we performed a small-scale functional siRNA screen of a panel of DUBs implicated in the regulation of HIF1α, including USP8, USP28, USP29, USP36, USP37, and UCHL1, with the Sorafenib-resistant HCC cell line HLE. siRNA-mediated knockdown of USP29 had the strongest effect on HIF1α protein levels, indicating that USP29 might be a key DUB in promoting HIF1α protein stability (Suppl. Fig. 3a, b). To further validate the role of USP29 in HIF1α stability, we analyzed the effect of two unique siRNAs against USP29 with varying knock-down efficiencies on HIF1α protein levels in Sorafenib-resistant HLE and SNU398 cells. Indeed, the efficiency of USP29 depletion of the two different siRNAs correlated with the extent of HIF1α protein loss (Fig. 2a; Suppl. Fig. 3c). Moreover, transfection of an increasing amount of plasmid expressing USP29 resulted in increasing stabilization of HIF1α in HLE cells (Fig. 2b). Finally, transfection of a siRNA-refractory cDNA encoding USP29 efficiently restored HIF1α protein levels in HLE and HEK-293T cells lacking endogenous USP29 (Fig. 2c; Suppl. Fig. 3d), supporting a role of USP29 in stabilizing HIF1α in HCC and HEK cells.

We further determined the robustness of USP29-mediated stabilization of HIF1α in a hypoxia-reoxygenation assay in Sorafenib-resistant HLE cells. While HIF1α levels were high under hypoxic culture conditions as compared with normoxia, upon reoxygenation HIF1α protein levels diminished over time in siControl-transfected cells (Fig. 2d). However, upon siRNA-mediated depletion of USP29, the loss of HIF1α protein was substantially accelerated, further supporting the key role of USP29 in stabilizing HIF1α.

As a transcription factor, HIF1α has to translocate to the nucleus to mediate its transcriptional outputs. We thus investigated the functional impact of USP29 on HIF1α nuclear localization and transcriptional activity. First, analysis of HLE cells by immunofluorescence microscopy revealed an increase in nuclear localization of HIF1α upon expression of Myc-tagged USP29 (Fig. 2e) and a reduction of nuclear HIF1α upon siRNA-mediated depletion of USP29 (Fig. 2f; Suppl. Fig. 3e). Second, the expression of HIF1α target genes was significantly increased upon the forced expression of Myc-tagged USP29, while the mRNA levels of HIF1α remained unaffected (Fig. 2g). Conversely, siRNA-mediated depletion of USP29 expression in HLE cells reduced expression of HIF1α target genes (Fig. 2h). Third, in line with the observed effects of USP29 on the expression of HIF1α target genes, siRNA-mediated ablation of USP29 reduced HIF1α transcriptional activity comparable to the siRNA-mediated depletion of HIF1α itself, as determined by a hypoxia response element (HRE)-driven luciferase reporter assay (Fig. 2i). Together, these findings suggest that USP29 is a potent positive regulator of HIF1α protein stability and transcriptional activity in Sorafenib-resistant HCC cells.

### USP29 interacts with and deubiquitylates HIF1α

**Fig. 3:**
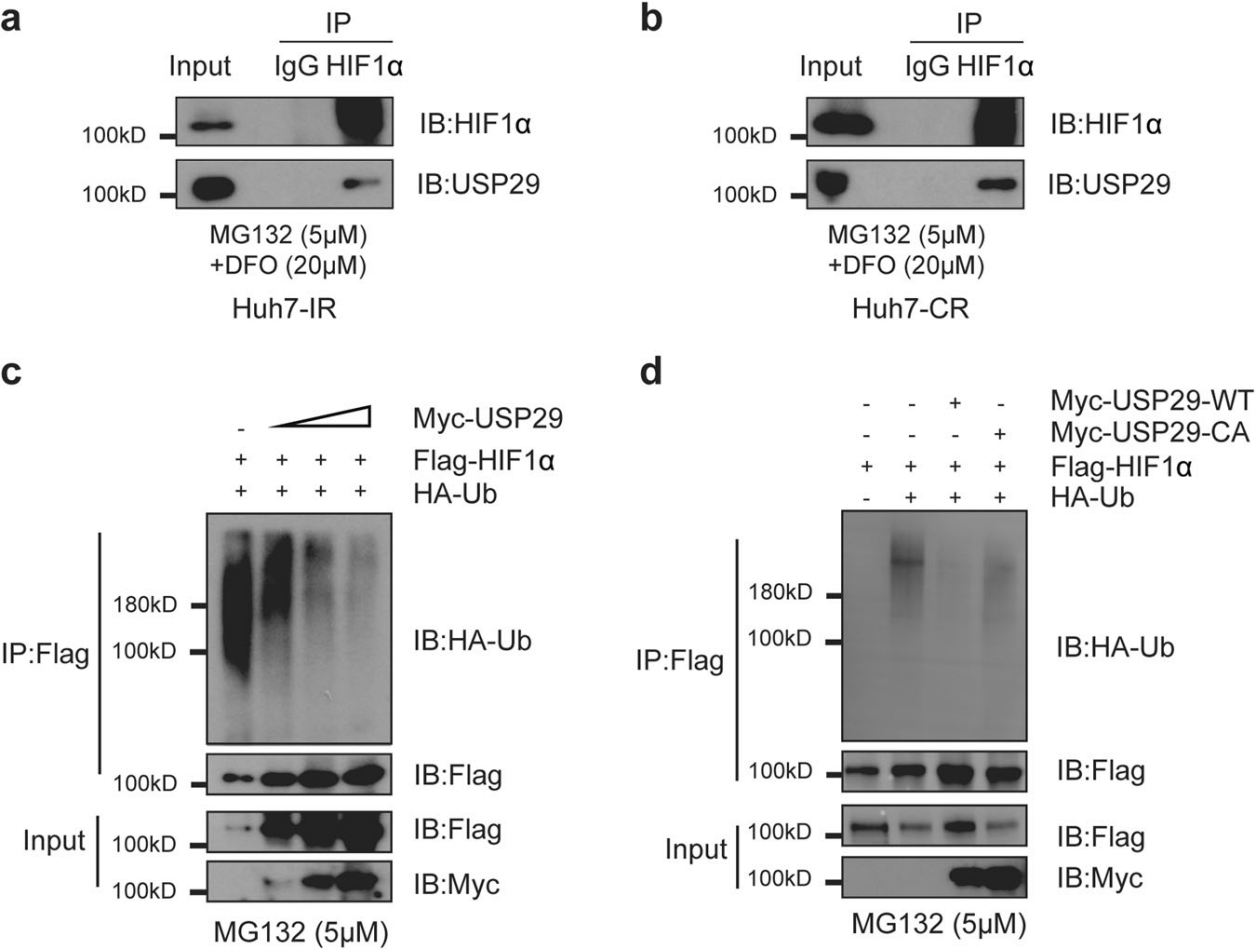
USP29 interacts with and deubiquitylates HIF1α. **a**, **b** Endogenous HIF1α interacts with USP29 in Sorafenib-resistant Huh7-IR and Huh-CR cells. Huh7-IR (**a**) and Huh-CR (**b**) cells were treated with 5 µM MG132 and 20 µM DFO for 8 h before harvest to enrich for HIF1α protein. anti-HIF1α antibody and irrelevant anti-IgG as control were used to precipitate (IP) endogenous HIF1α. Immunoprecipitates were then immunoblotted (IB) for HIF1α and for USP29. Input represents 1/10 of the lysate used for the immunoprecipitations. Results represent three independent experiments. **c** USP29 removes poly-ubiquitin from HIF1α. Increasing amounts of a plasmid encoding for Myc-USP29 were transfected together with Flag-HIF1α and HA-tagged ubiquitin (HA-Ub) into HEK-293T cells. Flag-HIF1α was then immunoprecipitated (IP) with anti-Flag antibody, and the precipitates were immunoblotted (IB) for HA (HA-Ub) and for Flag (Flag-HIF1α). USP29 reduced the poly-ubiquitination level of HIF1α. Input represents 1/10 of the lysate used for the immunoprecipitations. 5 µM MG132 was added into the culture medium 8 h before harvest to prevent proteasomal degradation. Results represent three independent experiments. **d** The catalytic activity of USP29 is required to remove poly-ubiquitin from HIF1α and to stabilize it. Plasmids encoding for Myc-tagged wildtype USP29 or Myc-tagged CA-mutant USP29 were transfected together with Flag-HIF1α and HA-tagged ubiquitin (HA-Ub) into HEK-293T cells. HIF1α was then immunoprecipitated (IP) with anti-Flag antibody, and the precipitates were immunoblotted (IB) for HA (HA-Ub) and for Flag (Flag-HIF1α). USP29 reduced the poly-ubiquitination level of HIF1α. Input represents 1/10 of the lysate used for the immunoprecipitations. 5 µM MG132 was added into the culture medium 8 h before harvest to prevent proteasomal degradation. Results represent three independent experiments.

We next sought to investigate the molecular mechanisms underlying USP29-mediated regulation of HIF1α protein stability. We hypothesized that USP29, as a DUB, specifically deubiquitylated HIF1α, thereby preventing its proteasomal degradation. To this end, we first examined a physical interaction between USP29 and HIF1α. Indeed, we found that USP29 binds HIF1α in both Huh7-IR and Huh7-CR Sorafenib-resistant cells (Fig. 3a, b). We also found that exogenous USP29 and HIF1α interact with each other when expressed in HEK-293T cells (Suppl. Fig. 3f, g). Moreover, exogenously expressed USP29 physically interacts with endogenous HIF1α in intrinsically Sorafenib-resistant HLE cells (Suppl. Fig. 3h).

Next, we assessed the functional consequence of the interaction of USP29 and HIF1α by focusing on the ubiquitination status of HIF1α. Interestingly, we observed less ubiquitylation of HIF1α upon expression of USP29 in a dose-dependent manner (Fig. 3c). The removal of ubiquitin on HIF1α seemed to be directly catalyzed by USP29, since a catalytically inactive form of USP29 (CA) failed to reduce ubiquitination of HIF1α (Fig. 3d) and, as a consequence, the CA form of USP29 was unable to stabilize HIF1α. Together, these results demonstrate that USP29 interacts with HIF1α to de-ubiquitinate and stabilize it.

### USP29 is a regulator of Sorafenib-resistance

**Fig. 4:**
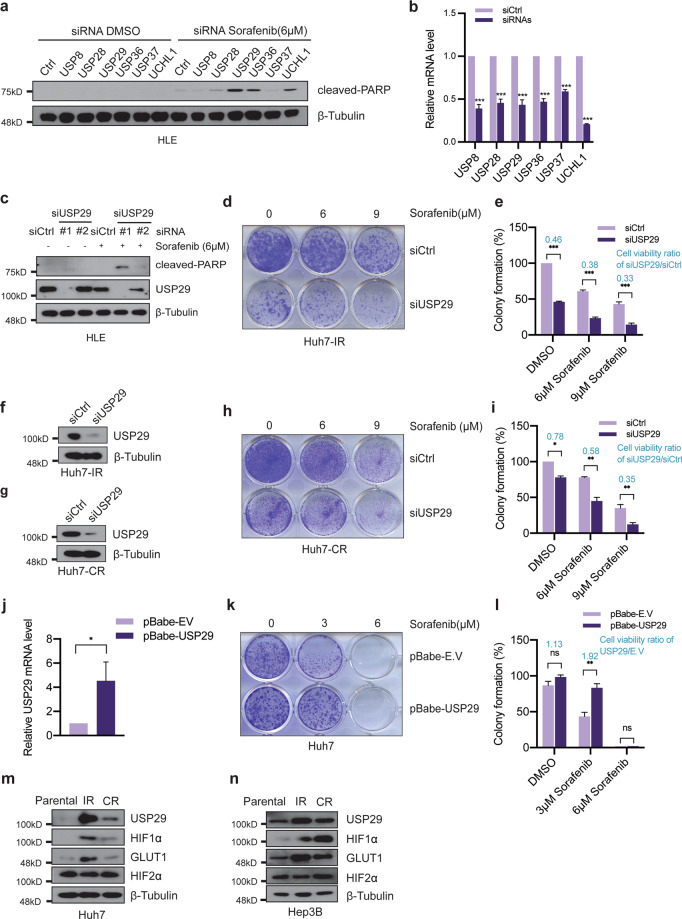
USP29 is a regulator of Sorafenib resistance in HCC. **a** siRNA-mediated mini-screen to identify DUBs required for cell survival. USP29 deficiency induces the highest levels of HLE cell apoptosis in response to short-term Sorafenib treatment. ON-TARGET siRNAs against selected DUBs and siCtrl (ON-TARGET plus NON-TARGETing pool; Horizon Discovery) were transfected into HLE cells, and the cells were treated with DMSO or with 6 µM Sorafenib, respectively, for 18 h. Immunoblotting for cleaved PARP shows that the depletion of USP29 induced the highest levels of apoptosis compared with other siRNAs. Immunoblotting for β-Tubulin was used as loading control. **b** Knockdown efficiencies of the siRNAs used in (**a**). Different siRNAs targeting USP8, USP28, USP29, USP36, USP37, UCHL1 were transfected into HLE cells, and quantitative RT-PCR analysis were conducted to determine knock down efficiencies. Results represent three independent experiments. **c** Two distinct siRNAs against USP29 (siUSP29#1 and siUSP29#2) were transfected into HLE cells, and the cells were treated with DMSO or 6 µM Sorafenib, respectively, for 18 h. Immunoblotting shows that siUSP29#1 had more knock down efficiency than siUSP29#2, and that the extent of cleaved PARP as a measure for apoptosis increased with knockdown efficiency. Immunoblotting for β-Tubulin was used as loading control. Results represent three independent experiments. **d**–**i** USP29 deficiency represses cell growth in Sorafenib-resistant cells. Colony formation assay was performed with Huh7-IR (**d**) and Huh7-CR cells (**h**) transfected with either siCtrl or ON-TARGET siUSP29 and treated with increasing concentrations of Sorafenib (0 μM, 6 μM, 9 μM) for two weeks. Colony formation was quantified by measuring crystal violet staining (**e**, **i**). The ratio of cell viability between USP29-deficient and USP29-wildtype cells is given in blue numbers (**e**, **i**). *n* = 3 independent replicates. ns = not significant; *P < 0.05; **P < 0.01; ***P < 0.001; Student’s *t*-test. The efficiency of USP29 knockdown in Huh7-IR and Huh7-CR cells was validated by immunoblotting (**f**, **g**). β-Tubulin was used as loading control. Results represent three independent experiments. **j**–**l** USP29 overexpression promotes cell survival in Sorafenib-sensitive cells (Huh7). Empty vector and USP29 stable-overexpressed Huh7 cell lines were established by the infection with empty lentivirus (pBabe-EV) or lentivirus coding for USP29 (pBabe-USP29). Quantitative RT-PCR analysis was conducted to validate the overexpression of USP29 (**j**). *n* = 2 independent replicates. Growth of the infected cells was determined by colony formation assay with Huh7-pBabe-EV and Huh7-pBabe-USP29 cells upon treatment with increasing concentrations of Sorafenib (0, 3, 6 μM) for two weeks (**k**). Colony formation was quantified by crystal violet staining (**l**). The ratio of cell viability between USP29-overexpressing and USP29-wildtype cells is given in blue numbers (**l**). *n* = 3 independent replicates. ns not significant; *P < 0.05; **P < 0.01; ***P < 0.001; Student’s *t*-test. **m**, **n** Immunoblotting analysis revealed that USP29 and HIF1α and its target GLUT1 were specifically expressed in Sorafenib-resistant Huh7-IR and Huh7-CR cells (**m**) and Hep3b-IR and Hep3B-CR cells (**n**), as compared to their parental Sorafenib-responsive cells, while HIF2α was not. Immunoblotting for β-Tubulin was used as loading control. Results represent three independent experiments.

The above results prompted us to further delineate the functional contribution of the USP29-HIF1α axis to Sorafenib resistance in HCC cells. We first tested whether the depletion of well-known DUBs for HIF1α, including USP8, USP28, USP29, USP36, USP37, and UCHL1, exert a synthetic lethal effect on Sorafenib-resistant HLE cells in the presence of Sorafenib. As a functional readout, we monitored the level of cancer cell apoptosis by immunoblotting for cleaved PARP. In line with our findings described above, siRNA-mediated knockdown of USP29 led to the highest levels of cell death in combination with Sorafenib treatment, compared to knockdown of other DUBs (Fig. 4a–c). Similar results were obtained with Hep3B and Huh7 cells, indicating that USP29 exerts a critical and general role in mediating Sorafenib resistance in HCC cells (Suppl. Fig. 4a, b).

To further explore the impact of USP29 in Sorafenib-resistant cells, we performed long-term colony formation assays with Huh7-IR and Huh7-CR cells. Indeed, we found that siRNA-mediated knockdown of USP29 reverted the acquired Sorafenib resistance in Huh7-IR and Huh7-CR cells and also the intrinsic Sorafenib resistance in HLE, SNU398, SNU449, and SNU475 cells (Fig. 4d–i; Suppl. Fig. 4c–n). Notably, the Sorafenib sensitivity induced by siRNA-mediated ablation of USP29 could be overcome by hypoxia treatment (Suppl. Fig. 5a–d). Conversely, stable overexpression of USP29 confers resistance to Sorafenib to otherwise Sorafenib-sensitive Huh7 cells (Fig. 4j–l). Analysis of the expression of USP29 revealed an increase in USP29 protein in Sorafenib-resistant Huh7-IR/CR and Hep3B-IR/CR cells as compared to their Sorafenib-sensitive parental cells (Fig. 4m, n). Consistent with increased USP29 protein levels, we observed an upregulation of HIF1α and HIF1α target genes (Fig. 4m, n). Together, the above results demonstrate a general role of the USP29-HIF1α axis in driving Sorafenib resistance in HCC cells. The molecular mechanisms underlying the stabilization of USP29 protein remain to be investigated.

### The USP29-HIF1α axis promotes glycolysis to mediate Sorafenib resistance

**Fig. 5:**
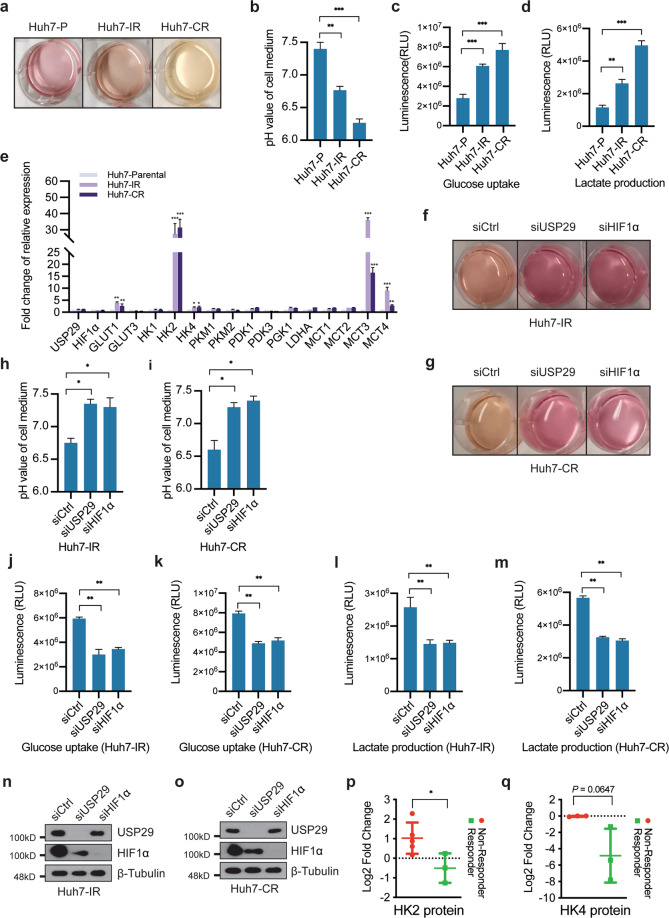
USP29/HIF1α axis in the regulation of glycolysis. **a**, **b** Huh7-IR/CR cells present increased acidification of cell medium. Cells were plated at the same numbers, and color changes of the culture medium were recorded 24 h after the seeding (**a**). pH values of the culture media were directly measured (**b**). *n* = 3 independent replicates. **P < 0.01; ***P < 0.001; Student’s *t*-test. **c**, **d** Sorafenib-resistant cells present with levels of glycolysis. Glucose uptake (**c**) and lactate production (**d**) were determined in Sorafenib-responsive Huh7 parental cells and in Sorafenib-resistant Huh7-IR and Huh7-CR cells. Normalized to cell numbers, Huh7-IR/CR cells showed higher glucose uptake and lactate production levels than Huh7 parental cells. *n* = 2 independent replicates. **P < 0.01; ***P < 0.001; Student’s *t*-test. **e** The mRNA levels of a selected subset of glycolysis-related genes were determined by quantitative RT-PCR in Sorafenib-responsive Huh7 parental cells and in Sorafenib-resistant Huh7-IR and Huh7-CR cells. High transcriptional levels of *GLUT1*, *HK2*, *HK4*, *MCT3,* and *MCT4* were found in the Sorafenib-resistant cells. The expression of *USP29* and *HIF1α* was unchanged between Huh7 parental cells and Huh7-IR/CR cells. *n* = 3 independent replicates. ns not significant; **P < 0.01; ***P < 0.001; Student’s *t*-test. **f**–**i** Depletion of USP29 or HIF1α diminishes the acidification of the culture medium in Sorafenib-resistant cells. Huh7-IR (**f**) and Huh7-CR (**g**) cells were plated at the same cell numbers 24 h after the transfection with siCtrl or ON-TARGET siRNAs against USP29 and HIF1α. Color changes were recorded 24 h later (**f**, **g**). pH values of the culture medium were directly measured in Huh7-IR (**h**) and Huh7-CR (**i**) cells. *n* = 2 independent replicates. *P < 0.05; Student’s *t*-test. **j**–**m** USP29 or HIF1α deficiency reduces glycolysis metabolism in Sorafenib-resistant cells. Huh7-IR (**j**, **l**) and Huh7-CR (**k**, **m**) cells were plated at the same cell numbers and transfected with siCtlr or ON-TARGET siRNAs against USP29 and HIF1α. Glucose uptake (**j**, **k**) and lactate production (**l**, **m**) were examined by determining relative luminescence (RLU) levels 24 h after siRNA transfection and normalized to cell numbers. *n* = 2 independent replicates. **n**, **o** Knockdown efficiencies of siRNAs against USP29 and HIF1α used in (**j**–**m**) as determined by immunoblotting. **p**, **q** Protein levels of hexokinase 2 (HK2), a major enzyme of the glycolytic pathway (**p**), and of hexokinase 4 (HK), the major liver hexokinase (**q**), were determined in a database of the whole proteomic analysis of needle biopsies from patients with HCC who responded to Sorafenib treatment (responder) or did not respond (non-responder). *P < 0.05; Student’s *t*-test.

Besides the hypoxia response, the transcriptomic analysis of Sorafenib-resistant cells also revealed pathways involved in cellular metabolism (Fig. 1a). In this context, we observed that the color of the culture medium of Sorafenib-resistant cells quickly changed to yellow even when cultured in the absence of Sorafenib (Fig. 5a). This observation suggested a general acidification caused by increased glycolysis and lactate excretion, and pH determination revealed lower pH values in the medium of Sorafenib-resistant Huh7-IR and Huh7-CR cells as compared to parental cells (Fig. 5b). To determine whether Sorafenib resistance is linked to glycolysis, we first measured glucose uptake and lactate production in Sorafenib-resistant Huh7-IR and Huh7-CR and in Sorafenib-responsive parental Huh7 cells. Indeed, we observed higher glucose uptake and lactate production in Sorafenib-resistant cells in comparison to their parental cells (Fig. 5c, d). In line with our transcriptomic analysis, a subset of glycolytic gene transcripts was found specifically upregulated in Sorafenib-resistant cells (Fig. 5e). These results indicate a glycolytic shift in Sorafenib-resistant cells.

It is widely recognized that glycolysis is an adaptive metabolic response driven by various stresses, such as hypoxia and drug therapy [19,20,]. HIF1α is one of the pivotal regulators of glycolysis by direct transcriptional regulation of key glycolytic genes. The finding that the USP29-HIF1α axis regulates Sorafenib resistance motivated us to investigate the functional connection between glycolysis and USP29-HIF1α-driven Sorafenib resistance. To this end, we first assessed the functional contribution of USP29-HIF1α to the acidification of culture medium. In comparison to siControl-transfected cells, siRNA-mediated depletion of USP29 or HIF1α in Sorafenib-resistant Huh7-IR and Huh7-CR cells prevented the culture medium color change and acidification (Fig. 5f–i) and reduced glucose uptake and lactate production (Fig. 5j–o).

We next assessed whether key glycolytic network gene transcripts were changed upon depletion of the USP29-HIF1α pathway in Sorafenib-resistant cells. Interestingly, the expression of *GLUT1*, *HK2*, *HK4*, *PDK1*, *MCT3*, and *MCT4* was significantly down-regulated in Sorafenib-resistant Huh7-IR and Huh-CR cells upon siRNA-mediated depletion of USP29 and HIF1α, compared to siControl-transfected cells (Suppl. Fig. 6a, b).

To assess whether Sorafenib resistance was linked to upregulation of glycolysis in HCC of patients, we analyzed the proteome of needle biopsies from tumors of Sorafenib responders and non-responders. Consistent with our in vitro analysis, Sorafenib non-responders showed high levels of HK2 in contrast to responders, indicating a potential upregulation of glycolysis in Sorafenib-resistant HCC of patients (Fig. 5p). The expression of the major liver hexokinase HK4 moderately, yet not significantly correlated with Sorafenib response in patients (Fig. 5q). Altogether, these findings suggest that USP29-mediated stabilization of HIF1α and its transcriptional output promote glycolysis and thus Sorafenib resistance in HCC cells.

### USP29 promotes sorafenib resistance in vivo

**Fig. 6:**
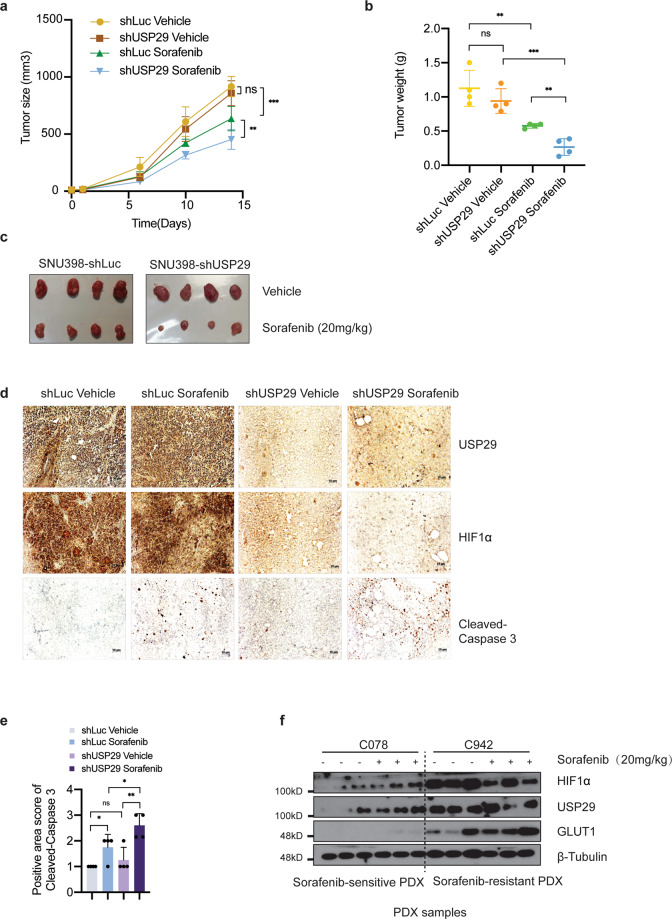
USP29 regulates responses to Sorafenib treatment in vivo. **a**–**c** Xenotransplanted HCC is re-sensitized to Sorafenib treatment upon depletion of USP29. Sorafenib-resistant SNU398 cells expressing either a control shRNA (shLuc) or a shRNA against USP29 (shUSP29) were implanted into the flanks of immunodeficient NSG mice and treated with vehicle solution or Sorafenib, respectively. Tumor growth curves over time (**a**) and tumor weights at the time of sacrifice (**b**) were determined, *N* = 4. Images of the tumors at the time of sacrifice are shown in (**c**). ns not significant; **P < 0.01; ***P < 0.001; Two-way ANOVA. **d**, **e** USP29-deficient tumors exhibit higher rates of apoptosis upon Sorafenib treatment. Histological sections of the tumors described in (**a**–**c**) were immunostained for USP29 and HIF1α (**d**). Immunostaining for cleaved Caspase 3 was used to quantify apoptosis (**d**, **e**). **f** Sorafenib-resistant PDX tumors present high USP29, HIF1α, and GLUT1 protein levels. Tumor pieces of HCC patient-derived xenotransplanted (PDX) mice which have been previously classified as Sorafenib-responsive or Sorafenib-resistant were analyzed by immunoblotting for the expression of HIF1α, USP29, and GLUT1. Immunoblotting for β-Tubulin was used as loading control. Results represent three independent experiments.

We next determined whether USP29 is required for Sorafenib resistance in vivo. To this end, Sorafenib-resistant SNU398 HCC cells were modified to stably express an shRNA against luciferase as control or an shRNA against USP29. These cells were then implanted into the flanks of immunodeficient NSG mice which were then treated or not with Sorafenib, and tumor growth was monitored over time. In line with our in vitro observations, tumor growth was significantly delayed and tumor weights significantly reduced upon USP29 knockdown and concomitant treatment with Sorafenib (Fig. 6a–c). This result indicated that depletion of USP29 re-sensitized Sorafenib-resistant HCC cells to Sorafenib in a preclinical mouse model of HCC in vivo. Immunohistochemical staining of USP29 and HIF1α on tumor sections confirmed that the siRNA-mediated knockdown of USP29 not only efficiently depleted USP29 but also reduced HIF1α levels (Fig. 6d). Immunohistochemical staining of cleaved Caspase 3 in tumor sections revealed that cancer cell apoptosis was increased by Sorafenib treatment but was even higher upon combination of Sorafenib with depletion of USP29 (Fig. 6d, e).

Finally, to explore whether an increased activity of the USP29-HIF1α axis correlated with Sorafenib resistance in patient samples, we analyzed USP29 and HIF1α in patient-derived xenotransplanted (PDX) tumors which were previously classified as either sensitive or resistant to Sorafenib treatment [21]. Indeed, immunoblotting revealed high levels of USP29, HIF1α, and its transcriptional target GLUT1 in Sorafenib-resistant PDX tumors as compared to Sorafenib-sensitive PDX tumors (Fig. 6f). Notably, the levels of these proteins were also increased in Sorafenib-sensitive tumors upon acute Sorafenib treatment, while the levels were already very high even without Sorafenib treatment in Sorafenib-resistant tumors.

In conclusion, the above data uncovered USP29 as a new regulator of HIF1α transcriptional activity which is critical to maintain Sorafenib-resistance in HCC cells by promoting glycolysis. Hence, the USP29-HIF1α axis represents a potential therapeutic target to overcome Sorafenib resistance in HCC.

## Discussion

Sorafenib, a small molecule multi-kinase inhibitor, targets Raf-1, B-Raf, vascular endothelial growth factor receptors (VEGFRs) [2,22,], and PDGFR-β (platelet-derived growth factor receptor β) involved in cancer cell proliferation, angiogenesis, and invasion in a wide range of cancer cells [23,24,]. It is the first line of standard therapy that has been approved by the FDA in 2007 for the treatment of advanced HCC patients. However, based on the sobering observation that the targeted therapy with Sorafenib has only a moderate and transient effect on HCC progression and fails to cure HCC patients, the delineation of the molecular mechanisms underlying Sorafenib resistance and the design and development of alternative therapies overcoming Sorafenib resistance are important.

Several factors and signaling pathways have been reported previously to contribute to Sorafenib resistance, including the PI3K-AKT, JAK-STAT, and ERK2 signaling pathways, epithelial-mesenchymal transition, and hypoxia-induced signaling [25,26,]. Previous results from our laboratory have identified LATS1 as a regulator of Sorafenib resistance in mediating a cross-talk between Hippo signaling and autophagy [18]. In spite of these findings, the actual mechanisms of Sorafenib resistance and potential therapeutic targets to overcome it still remain widely elusive.

Here we have identified the deubiquitylating enzyme (DUB) USP29 as one critical player in the maintenance of Sorafenib resistance in HCC cells in vitro and in vivo. USP29 deubiquitylates HIF1α, thereby stabilizing and activating it. Hypoxia is a key microenvironmental factor promoting cancer progression, including the induction of Sorafenib resistance in several different cancer types [27,28,]. As a frequent feature of solid tumors, hypoxia promotes cancer cell proliferation, tumor angiogenesis, metastasis, and metabolic changes which altogether may cause therapy resistance. HIFs are transcription factors that execute the response to oxygen deprivation. HIF1α and HIF2α have been reported to be highly expressed in HCC and both contribute to Sorafenib resistance [29,30,]. However, we found that HIF1α, but not HIF2α, is highly expressed in Sorafenib-resistant HCC cells. Moreover, depletion of HIF1α caused the death of Sorafenib-resistant cells, while depletion of HIF2α did not, indicating that HIF1α is the critical factor in maintaining Sorafenib resistance in HCC cells. These results are consistent with previous reports suggesting that HIF1α confers Sorafenib resistance in HCC patients [5,31,]. However, the actual mechanisms by which HIF1α activity is regulated in Sorafenib-resistant HCC cells and patients had remained unclear.

HIF1α protein stability is regulated by ubiquitination. In the presence of sufficient oxygen, HIF1α is ubiquitylated by the E3 ligase VHL and then rapidly degraded by the UPS [6–10]. Conversely, DUBs such as USP8, USP28, UCHL1 stabilize HIF1α by removing the polyubiquitin of HIF1α upon hypoxia or normoxia [6,32,33,]. Employing a functional mini-screen of a selected subset of DUBs, we identified USP29 as the most critical DUB in the stabilization and activation of HIF1α and in supporting Sorafenib resistance in HCC cells.

The contribution of DUBs to tumor progression and therapy resistance is not without precedence. For example, USP28 has been reported to stabilize MYC and to be highly expressed in colon and breast cancers [34]. USP36 and USP37 have been reported to regulate tumorigenesis by preventing MYC degradation in breast and lung cancer [16,35,]. USP7 can stabilize MDM2 to prevent degradation of the tumor suppressor p53 [36,37,], and USP8 has been described as a novel target for overcoming Gefitinib resistance in lung cancer [38]. In the context of HCC, USP10 promotes HCC cell proliferation and metastasis by deubiquitinating and stabilizing YAP and TAZ, the effector transcription factors of the Hippo signaling pathway, and SMAD4, the major signaling effector of TGFβ signaling [39,40,]. Thus, small molecular inhibitors have been developed to interfere with DUB function. P5091 (inhibitor of USP7) and b-AP15 (inhibitor of USP14/UCHL5) inhibit the growth of bortezomib-resistant multiple myeloma [41,42,]. The USP8 inhibitor 9-ethyloxyimino-9H-indeno[1,2-b] pyrazine-2,3-dicarbonitrile suppresses growth of non-small cell lung carcinoma (NSCLC) cells [38]. Here, we report that depletion of USP29 is sufficient to re-sensitize HCC cells to Sorafenib in vitro and in vivo, suggesting that USP29 is a novel and suitable target for overcoming Sorafenib resistance in HCC.

Consistent with previous reports [43,44,], our transcriptomic analysis of Sorafenib-resistant HCC cells revealed that glycolysis was highly upregulated in Sorafenib-resistant cells. Our study demonstrates that USP29 is associated with glycolysis through HIF1α, including glucose uptake and lactate production. Depletion of USP29 significantly reduces glycolysis and lactate production. Downregulation of *GLUT1*, *HK2*, *HK4*, *MCT3*, and *MCT4* upon USP29 knockdown further supports a link between USP29 and glycolysis. Notably, high expression of HK2, but not of HK4, the major liver HK, significantly correlated with patients who did not respond to Sorafenib therapy. However, we note that the increased glycolysis observed in Sorafenib-resistant HCC cells may also be due to the ongoing cell growth and proliferation and the subsequent high energy demand of Sorafenib-resistant cells. Hence, upregulated glycolysis may be part of, but not the exclusive mechanism of promoting Sorafenib therapy resistance in HCC cells.

A high level of glycolysis has been shown to contribute to therapy resistance in different types of cancers. Our laboratory previously reported that resistance to anti-angiogenic therapy relies on a glycolytic shift that establishes a metabolic symbiosis between hypoxic, glycolytic, and lactate-producing tumor cells and normoxic, lactate-importing tumor cells which use lactate and oxygen for oxidative phosphorylation [45]. In this previous study, interference with glycolysis or lactate transport overcame therapy resistance, suggesting that interference with glycolytic pathways may contribute to overcoming Sorafenib resistance, a notion that warrants further investigation.

In summary, our study identifies USP29 as a novel DUB that stabilizes and activates the transcription factor HIF1α in HCC. This USP29-HIF1α axis induces a glycolytic shift in HCC cells which is coupled with Sorafenib resistance. Our study also suggests that USP29 and HIF1α are translational biomarkers for the prediction of therapy response in HCC patients, highlighting the USP29-HIF1α-glycolysis regulatory network as an emerging therapeutic target to overcome therapy resistance in HCC patients.

## Materials and methods

### DNA constructs, siRNAs, and antibodies

A cDNA construct encoding for Flag-HIF1α was amplified from a cDNA library and cloned into pcDNA4.0, and Myc-USP29 was amplified from a cDNA library and cloned into pcDNA4/TO/myc-His B. To generate pBabe-USP29, a cDNA fragment coding for USP29 was cloned into pBabe-retro-puro-empty vector. To generate Myc-USP29-CA, Myc-USP29 was mutated at C294S and H831N. pGL4.42 and pRL-CMV were purchased from Promega. On-target siRNAs were purchased from Horizon Discovery. siUSP29#1, siUSP29#2 were ordered from Microsynth and are listed in Suppl. Table II. Myc-USP29-R was mutated on Myc-USP29 to be resistant to siUSP29#1, primers are listed in Suppl. Table II. The Sequences of siRNAs are presented in Suppl. Table II, antibodies used are listed in Suppl. Table III, and oligonucleotides are listed in Suppl. Table IV.

### Cell culture, transfection, and reagents

HEK-293T, SNU398 were obtained from American Type Culture Collection (ATCC), Huh7, HLE, Hep3B were kind gifts from L. Quagliata (Institute of Pathology, University Hospital Basel). All cell lines used in this study were tested for the absence of Mycoplasma contamination every two weeks.

Plasmids transfection into HEK293T cells were carried out using PEI (Polyethylenimine, Linear, MW 25000, Polysciences Catalog No. 23966-1), plasmids transfection into HCC cells were carried out with Lipofectamine 3000 (Invitrogen). siRNA transfections were carried out with Lipofectamine RNAiMAX (Invitrogen) according to the manufacturer’s instructions. pBabe-retro-puro or pSuper-retro-puro constructs were used for establishing stable knock-down and stable overexpressing cell lines, Platinium-A cells were used for retrovirus production, infections were performed using 8 μg/ml Polybrene.

### Dual-Luciferase report assay

Cells were seeded into 24-well plates, transfections of siRNAs were performed once cell confluence had reached 60%. Medium was changed after 8 h. Twenty-four hours later, pGL4.42 and pRL-CMV were transfected together into cells in a 10:1 mass ratio, and medium was changed after 8 h. Cells were washed with PBS twice, and *Firefly* luminescence and *Renilla* luminescence were measured using Dual-Luciferase report Assay Kit (Promega E1980) and a (Berthold Centro LB 960).

### Glucose uptake assay

Cells were seeded into 96-well plates (5000 cells per well), treated with DMSO or 6 μM Sorafenib, respectively, 18 h later cells were washed with PBS twice and Glucose uptake levels were measured using Glucose Uptake-Glo™ Assay Kit (Promega J1341) and a Berthold luminometer (Berthold Centro LB 960).

### L-Lactate assay

Cells were seeded into 96-well plate (5000 cells per well), treated with DMSO or 6 μM Sorafenib, respectively, 18 h later the medium was collected, and cells were washed with PBS twice, and the lactate levels of cell medium and cells were measured by using Lactate-Glo™ Assay Kit (Promega J5021) and a Berthold luminometer (Berthold Centro LB 960).

### Colony formation assay

Cells were seeded into 12-well plates (5000 cells per well) and cultured for 2 weeks, siRNAs were transfected every other day, culture medium with either DMSO or Sorafenib was exchanged every 24 h. Two weeks later cells washed with PBS and fixed with 4% Paraformaldehyde for 30 min at room temperature, washed with PBS again and stained with crystal violet (1 mg/ml dissolved into 10% Ethanol) for 30 min at room temperature. After washing with PBS, plates were left to dry, and cells stained with crystal violet were counted using Fiji (NIH Image).

### Tumor transplantation

SNU398-shLuc or SNU398-shUSP29 cells (1 × 10^6^ in 100 μl PBS) were implanted into the left flanks of immuno-deficient NOD/SCID; common γ receptor-/- (NSG) mice. When tumors were palpable, vehicle solution or Sorafenib (20 mg/kg) was applied daily via gavage for 3 weeks. Tumor width and length were measured twice a week, tumor volumes were calculated using the formulation of volume = length × width^2 ^× 0.52. All animal experiments were performed according to the Swiss Federal Animal Welfare Law under approval number 2839 by the Veterinary Office of the Canton Basel Stadt.

### PDX models

HCC needle biopsies from HCC patients were obtained at the University Hospital Basel, Basel, Switzerland and implanted into NOD/SCID, common γ receptor-deficient (NSG) mice to establish patient-derived xenotransplantation mouse models of HCC as previously described [21]. Upon re-transplantation into the flanks of NSG mice and tumor palpation, the mice were treated with Sorafenib (20 mg/kg) for 5 weeks, growth curves were recorded and xenograft samples were collected for analysis. Experiments were conducted with the approval of the ethics committee of the northwestern part of Switzerland (protocol #EKNZ 2014‐099) and the animal care committee of Canton Basel‐Stadt, Switzerland.

### Protein lysis, immunoprecipitation, ubiquitination assay

For immunoblotting analysis, cells were washed with 1× PBS twice and lysed with RIPA lysis buffer (Sigma R0278). Cell lysates were centrifuged and the pellets were removed before protein concentration measurement and immunoblotting analysis.

For immunoprecipitation, cells were washed with 1× PBS twice and lysed with CST lysis buffer (CST9803) supplemented with protease inhibitors (Sigma P2714) at 4 °C, then centrifuged at 13000 rpm for 10 min, and pellets were removed. 1/10 of the cell lysate was taken as input, the rest of the cell lysate were incubated with specific antibodies and protein A/G-Sepharose overnight at 4 °C. After five times washing with CST lysis buffer, the precipitated proteins were eluted with SDS-loading buffer and analyzed by immunoblotting.

For ubiquitination assays, cells transfected with plasmids were lysed with RIPA buffer supplemented with an additional 0.1% SDS to a final concentration of 0.2% SDS, followed by standard immunoprecipitation protocols.

For immunoblotting analysis, protein samples were fractionated by SDS-PAGE gels and transferred to PVDF membranes, then membranes were blocked with 5% skimmed milk in TBST, and antibodies were incubated with the membranes overnight at 4 °C. Membranes were washed with TBST 3 × 10 min and incubated with the secondary antibodies for 2 h at room temperature, then washed for 3 × 10 min with TBST. Chemiluminescence was detected with X-Ray films or a Fusion device (Analis) once the membranes were incubated with chemiluminescent HRP substrate (Millipore WBKLS0500). Fiji software was used to quantify the immunoblots by densitometry (NIH Image). Information on the antibodies used is presented in Suppl. Table III.

### RNA extraction and real-time PCR

RNA samples were extracted with TRIZOL reagent (Sigma T9424), reverse transcription PCR was performed with Reverse Transcriptase kit (Promega A3803), real-time PCR was performed using Powerup SYBR Green PCR master mix (A25743) and a Step-One Plus real-time PCR machine (Applied Biosystems). Human RPL19 expression was used for normalization. Sequences of primers are listed in Suppl. Table IV.

### Immunofluorescence

Cells were cultured on coverslips, washed with PBS twice and fixed with 4% paraformaldehyde for 10 min, and then washed twice PBS. Cells were permeabilized with 0.1% Triton (DAPI was also diluted into Triton at 100 ng/ml to stain the nucleus) on ice for 10 mins. After three times wash with PBS, cells were blocked with 5% goat serum for 1 h at room temperature, then incubated with diluted antibodies (in 5% goat serum) overnight at 4 °C. Cells were washed with PBS three times then incubated with secondary antibody (1:200 dilution) at room temperature for 1 h. Then cells were washed with PBS three times, and mounting medium was added to mount coverslips to glass slides. Immunofluorescence staining was visualized on a Leica DMI 4000/6000 fluorescence microscope.

### Immunohistochemistry

Tumor sections were deparaffinized with 3×10 min Roticlear, 2 × 5 min 100% EtOH, 1 × 10 min 90% EtOH, 1×5 min 80% EtOH, 1×5 min 70% EtOH, 1 × 5 min 30% EtOH, 3 × 10 min PBS. Antigen retrieval was performed in 10 mM pH6.0 citrate buffer in a pressure cooker, wash 3 × 10 min with 0.3% Trition-100 in PBS. Peroxidase was quenched with 3% H_2_O_2_ for 10 min, followed by washing 3×10 min PBS, and blocking with 2.5% goat serum for 30 min at room temperature. Incubation with primary antibody (diluted into 2.5% goat serum) was at 4 °C overnight, followed by washing 3 × 10 min with PBS, incubation with secondary antibody (Vector MP-7541-50) at room temperature for 30 min, washing 3 × 10 min with PBS, incubation with peroxidase substrate (Vector SK-4105) at room temperature for 5 min and washing with water for 5 min. Counterstaining with Hematoxylin was done for 1 min to stain nuclei, followed by washing with water for 5 min, and dehydration with 50% EtOH, 70% EtOH, and 95% EtOH for 5 min each, then 2 × 10 min 100% EtOH, and clearing with 2 × 10 min xylene. Coverslips were mounted with 2–3 drops mounting media (Thermo Fisher Scientific Cytoseal™ XYL mounting media 8312-4) and let try overnight.

Slides were imaged with a Zeiss brightfield microscope (Zeiss Axioskop 2 Plus) and analyzed with Fiji (NIH Image). Positive area scores were defined as: (1) 0-25% positive area, (2) 26-50% positive area, (3) 51–75% positive area, (4) 76–100% positive area.

### RNA-sequencing analysis

RNA was extracted in biological triplicates using miRNeasy Mini kit (Qiagen) according to the manufacturer’s instructions. RNA quality control was performed using a fragment analyser and the standard or high-sensitivity RNA analysis kits (Labgene; DNF-471-0500 or DNF-472-0500). RNA concentrations were measured using the Quanti-iTTM RiboGreen RNA assay Kit (Life Technologies/Thermo Fisher Scientific). A total of 200 ng of RNA was utilized for library preparation with the TruSeq stranded total RNA LT sample prep Kit (Illumina). Poly-A + RNA was sequenced with HiSeq SBS Kit v4 (Illumina) on an Illumina HiSeq 2500 using protocols defined by the manufacturer.

Single-end RNA-seq reads (81-mers) were mapped to the human genome assembly, version hg19 (GRCh37.75), with RNA-STAR [46], with default parameters except for allowing only unique hits to the genome (outFilterMultimapNmax = 1) and filtering reads without evidence in spliced junction table (outFilterType = “BySJout”). Expression levels per gene (counts over exons) for the RefSeq mRNA coordinates from UCSC (genome.ucsc.edu, downloaded in December 2015) were quantified using qCount function from QuasR package (version 1.12.0). The differentially expressed genes were identified using the edgeR package (version 3.14.0). Genes with p-values smaller than 0.05 and minimum log2-fold changes of ±0.58 were considered as differentially regulated and were used for downstream functional and pathway enrichment analysis.

### Functional enrichment analysis

We performed functional enrichment analysis of differentially expressed genes for biological processes or pathways in R using several publicly available Bioconductor resources including org.Hs.eg.db (version 3.3.0), GO.db (version 3.4.1), GOstats (version 2.42.0) [47], KEGG.db (version 3.2.3) and ReactomePA (version 1.16.2) [48]. The significance of each biological process or pathway identified was calculated using the hypergeometric test (equivalent to Fisher’s exact test) and those with *p* values ≤0.05 were considered significant.

### Gene set enrichment analysis (GSEA)

The GSEA analysis was performed using the JAVA application of the Broad Institute version 3.0 (http://www.broadinstitute.org/gsea). The gene sets used for the analysis were derived from gene ontology annotations, and pathways were obtained from the Kyoto Encyclopedia of Genes and Genomes (KEGG) (http://www.genome.jp/kegg/) databases.

### Patient material and ethics

All relevant ethical regulations were strictly followed in this study. All the analyses using human tissue samples reported in this study were approved by the ethics commission of Northwestern Switzerland (EKNZ, approval No. 361/12).

### Statistical analysis

All statistical tests were two-sided. Data are presented as mean. Bar plots with error bars represent mean ± standard derivation (SD). Statistical significance is defined as *P < 0.05; **P < 0.01; ***P < 0.001. All analyses were performed using Prism 8.0 (Graphpad Software, Inc., La Jolla, CA).

## Supplementary information

Supplementary Information

Supplementary Table I

## Data Availability

Further information and requests for resources and reagents should be directed to and will be fulfilled by the Lead Contact, Gerhard Christofori (gerhard.christofori@unibas.ch). The RNA-sequencing files are deposited on GEO database under the accession number GSE158458.
