# Supplementary material for: USP29-mediated HIF1α stabilization is associated with Sorafenib resistance of hepatocellular carcinoma cells by upregulating glycolysis
Source: Oncogenesis. 2021 Jul 16;10(7):52. doi: 10.1038/s41389-021-00338-7 (PMC8285469; doi:10.1038/s41389-021-00338-7)
Supplement: Supplementary file 1 — Supplementary Information [file 41389_2021_338_MOESM1_ESM.pdf]

Supplementary Information

USP29-mediated HIF1α stabilization promotes Sorafenib resistance of hepatocellular carcinoma cells by upregulating glycolysis

Ruize Gao, David Buechel, Ravi K.R. Kalathur, Marco F. Morini, Mairene Coto-Llerena, Ercan Caner, Salvatore Piscuoglio, Qian Chen, Tanja Blumer, Xueya Wang, Eva Dazert, Markus H. Heim, Michael N. Hall, Fengyuan Tang, and Christofori Gerhard

Supplementary Figures

Suppl. Figure 1, Gao et al.

a

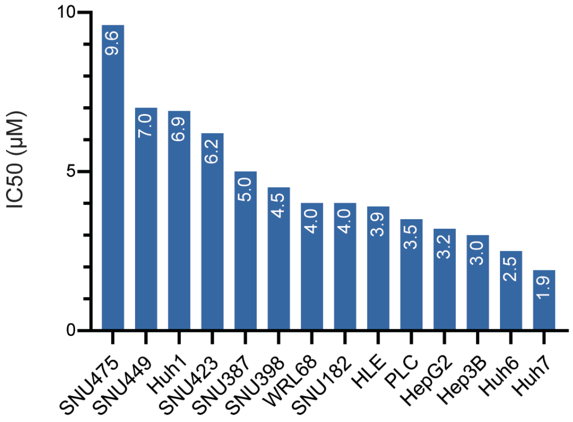

b

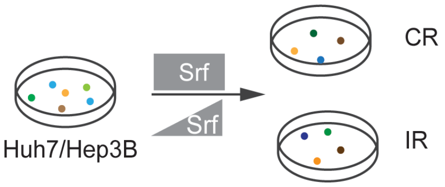

c

Cell line	IC50 (μM)
Huh7-IR	10.7
Huh7-CR	10.8
Hep3B-IR	7.2
Hep3B-CR	8.3

d

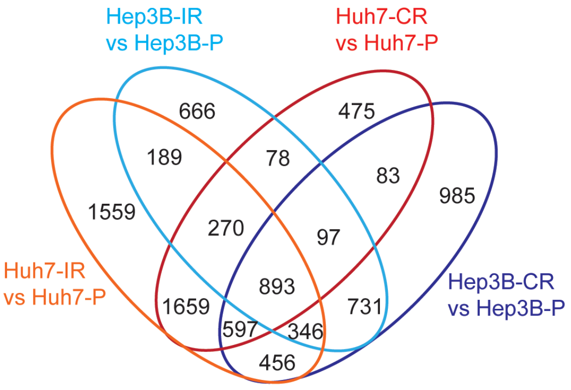

Supple. Figure 1. Establishment of Sorafenib-resistant cell lines and RNA sequencing.
(a) IC50 for Sorafenib responsiveness of different HCC cell lines. Different patient-derived HCC cell lines were treated with increasing doses of Sorafenib, and the IC50 values for cell growth inhibition by Sorafenib were determined. Hep3B and Huh7 were selected as two Sorafenib-responsive and HLE and SNU 398 as moderate Sorafenib-resistant HCC cell lines.
(b) Schematic representation of the establishment of Sorafenib-resistant derivatives of Huh7 and Hep3B HCC cell lines. Huh7 or Hep3B cells were treated with increasing or with consistently high concentration Sorafenib to establish acquired Sorafenib-resistant cell lines.
(c) Sorafenib IC50 values of Huh7-IR, Huh7-CR and Hep3B-IR and Hep3B-CR. These IC50 values are fairly close to Sorafenib's clinically relevant concentration of 10μM.
(d) Differential gene expression between the four Sorafenib-resistant cell lines and their Sorafenib-responsive ancestors. The Venn diagram shows the numbers of genes that are differentially expressed between individual parental and Sorafenib resistant cells and the number of genes which are changing with Sorafenib in several or all of the cell lines.

Suppl. Figure 2, Gao et al.

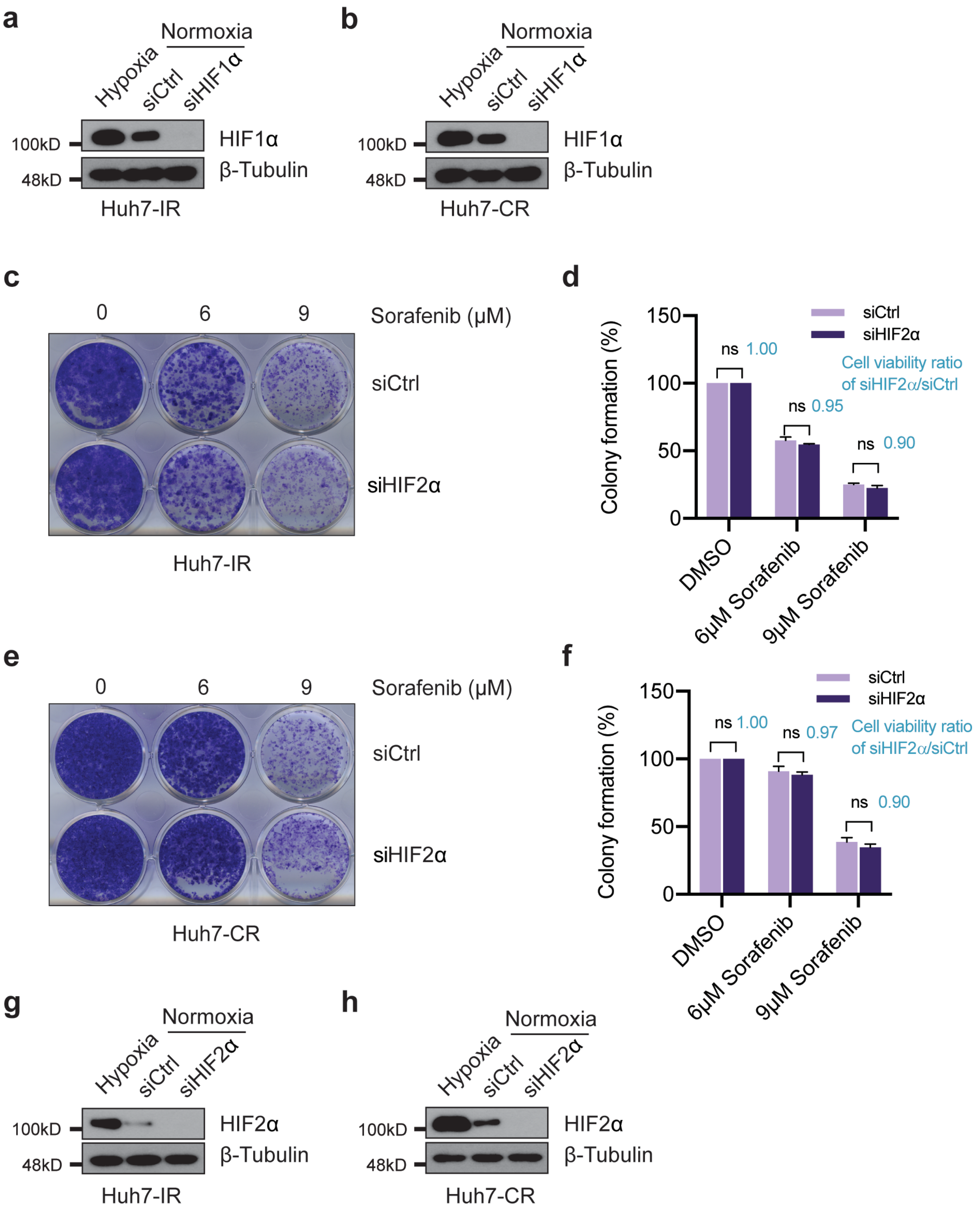

Suppl. Figure 2. HIF2α is not required for Sorafenib resistance.

(a,b) Validation of knockdown of HIF1α with ON-TARGET siHIF1α in Sorafenib-resistant Huh7-IR (a) and Huh7-CR (b) cells. Huh7-IR and Huh7-CR cells were transfected with siCtrl or ON-TARGET siHIF1α, and knockdown efficiency was validated by immunoblotting. Culture of the cells in hypoxia was used as a positive control for high HIF1α expression. β-Tubulin was used as loading control. Results represent three independent experiments.

(c-f) HIF2α's loss of function has no effect on cell death with Sorafenib treatment. Huh7-IR (c) or Huh7-CR (e) cells were transfected with siCtrl or siHIF2α and treated with DMSO or with 6μM or 9μM Sorafenib for 2 weeks. Colony formation assays (c,e) and quantification of colony formation by crystal violet staining (d,f) did not revealed any effect on colony formation by the siRNA-mediated depletion of HIF2α. The ratio of cell viability between HIF2α-deficient and HIF2α-wildtype cells is given in blue numbers (d,f). n = 3 independent replicates. ns = not significant; Student's t-test.

(g,h) Validation of knockdown of HIF2α with ON-TARGET siHIF2α in Huh7-IR (g) and Huh7-CR (h) cells. Huh7-IR and Huh7-CR cells were transfected with siCtrl or ON-TARGET siHIF2α, and knockdown efficiency was validated by immunoblotting. Culture in hypoxia was used as a positive control. β-Tubulin was used as loading control. Results represent three independent experiments.

Suppl. Figure 3, Gao et al.

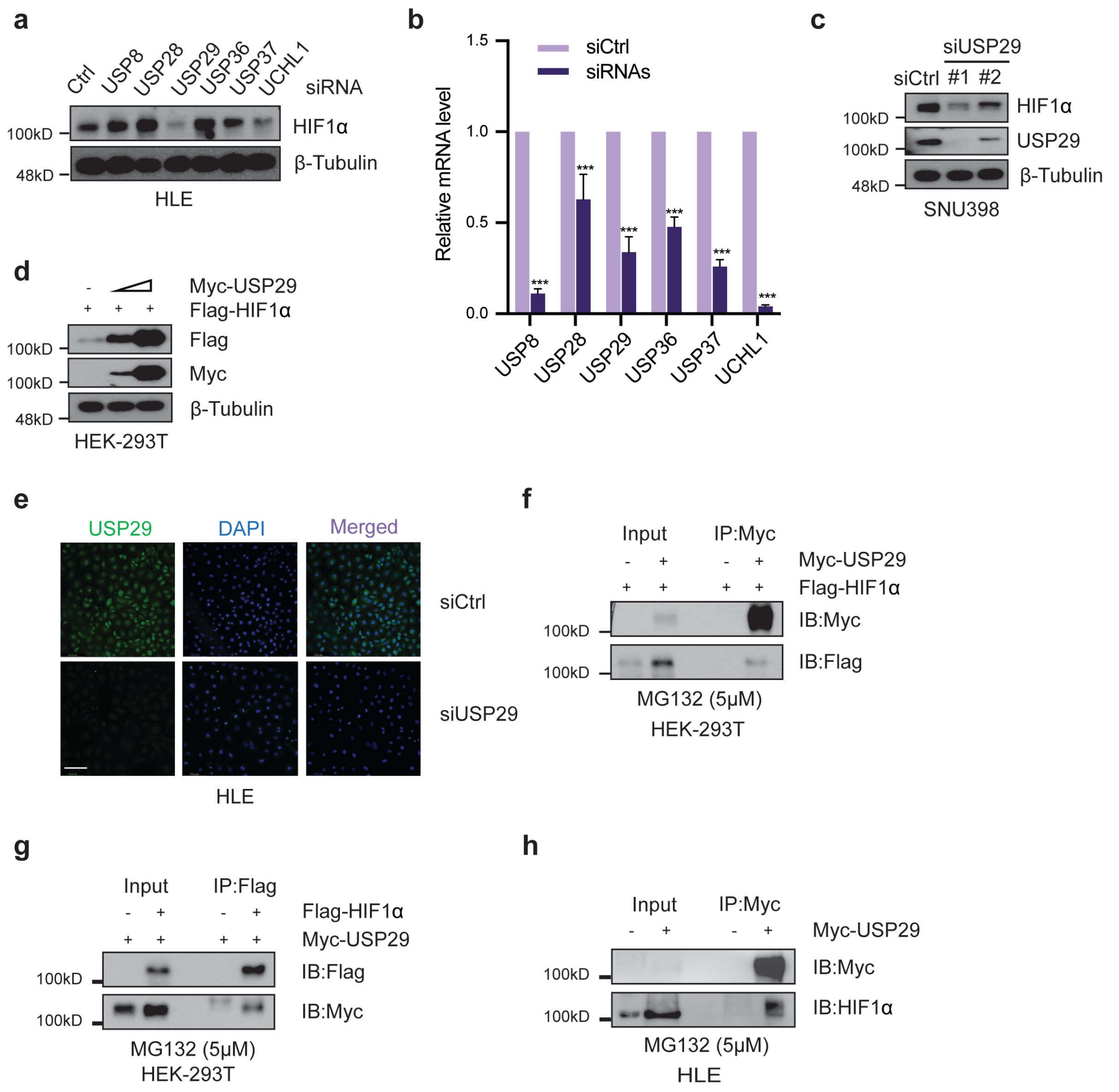

Suppl. Figure 3. USP29 has a positive correlation with HIF1α.

(a) Identification of USP29 as a DUB of HIF1α. A mini-screen with siRNAs against a selected panel of DUBs in Sorafenib-resistant HLE cells revealed that USP29 is one of the most critical players in stabilizing HIF1α protein, as revealed by immunoblotting for HIF1α. Immunoblotting for β-Tubulin was used as loading control. Results represent three independent experiments.

(b) Knockdown efficiencies of the siRNAs used in (a). Different ON-TARGET siRNAs targeting USP8, USP28, USP29, USP36, USP37, UCHL1 were transfected into HLE cells, and quantitative RT-PCR analysis were conducted to determine knock down efficiencies. n = 2 independent replicates. **, $P < 0.01$; ***, $P < 0.001$; Student's t-test.

(c) Loss of USP29 results in instability of HIF1α protein in Sorafenib-resistant SNU398 cells. Two distinct siRNAs against USP29 (siUSP29#1 and siUSP29#2) were transfected into SNU398 cells and the protein levels of HIF1α, and USP29 was determined by immunoblotting analysis. Immunoblotting for β-Tubulin was used as loading control. Results represent three independent experiments.
(d) USP29 stabilizes HIF1α. HEK-293T cells were transfected with a plasmid encoding for Flag-HIF1α together with increasing amounts of plasmid encoding for Myc-USP29. HIF1α and USP29 protein levels were determined by immunoblotting against Flag (Flag-HIF1α) and Myc (Myc-USP29). Immunoblotting for β-Tubulin was used as loading control. Results represent three independent experiments.
(e) Knockdown efficiency of siUSP29 expression. HLE cells were transfected with siCtrl and ON-TARGET siUSP29 and the expression and localization of USP29 was monitored by immunofluorescence microscopy analysis. DAPI was used to visualize nuclei. Results represent three independent experiments. Scale bar, 132.5μm.
(f) Myc-tagged USP29 binds Flag-tagged HIF1α. A plasmid encoding Flag-HIF1α was transfected into HEK-293T cells together with a plasmid encoding Myc-USP29. Anti-Flag antibodies were used to precipitate (IP) Flag-tagged HIF1α, and the immunoprecipitates were immunoblotted (IB) for Flag (Flag-HIF1α) and for Myc (Myc-USP29). Input represents 1/10 of the lysate used for the immunoprecipitations. Results represent three independent experiments. The proteasome inhibitor MG132 (5μM) was added in these experiments to prevent protein degradation.
(g) Flag-tagged HIF1α binds Myc-tagged USP29. A plasmid encoding for Myc-tagged USP29 was transfected into HEK-293T cells together with a plasmid coding for Flag-tagged HIF1α. Anti-Myc antibodies were used to precipitate (IP) Myc-tagged USP29, and the immunoprecipitates were immunoblotted (IB) for Myc (Myc-USP29) and for Flag (Flag-HIF1α). Input represents 1/10 of the lysate used for the immunoprecipitations. Results represent three independent experiments. The proteasome inhibitor MG132 (5μM) was added in these experiments to prevent protein degradation.
(h) Myc-tagged USP29 binds endogenous HIF1α. Plasmids encoding Myc-tagged empty vector or Myc-tagged USP29 were transfected into HEK-293T cells. Anti-Myc antibodies were used to precipitate (IP) Myc-tagged USP29, and the immunoprecipitates were immunoblotted (IB) for Myc (Myc-USP29) and for endogenous HIF1α. Input represents 1/10 of the lysate used for the immunoprecipitations. Results represent three independent experiments. The proteasome inhibitor MG132 (5μM) was added in these experiments to prevent protein degradation.

Suppl. Figure 4, Gao et al.

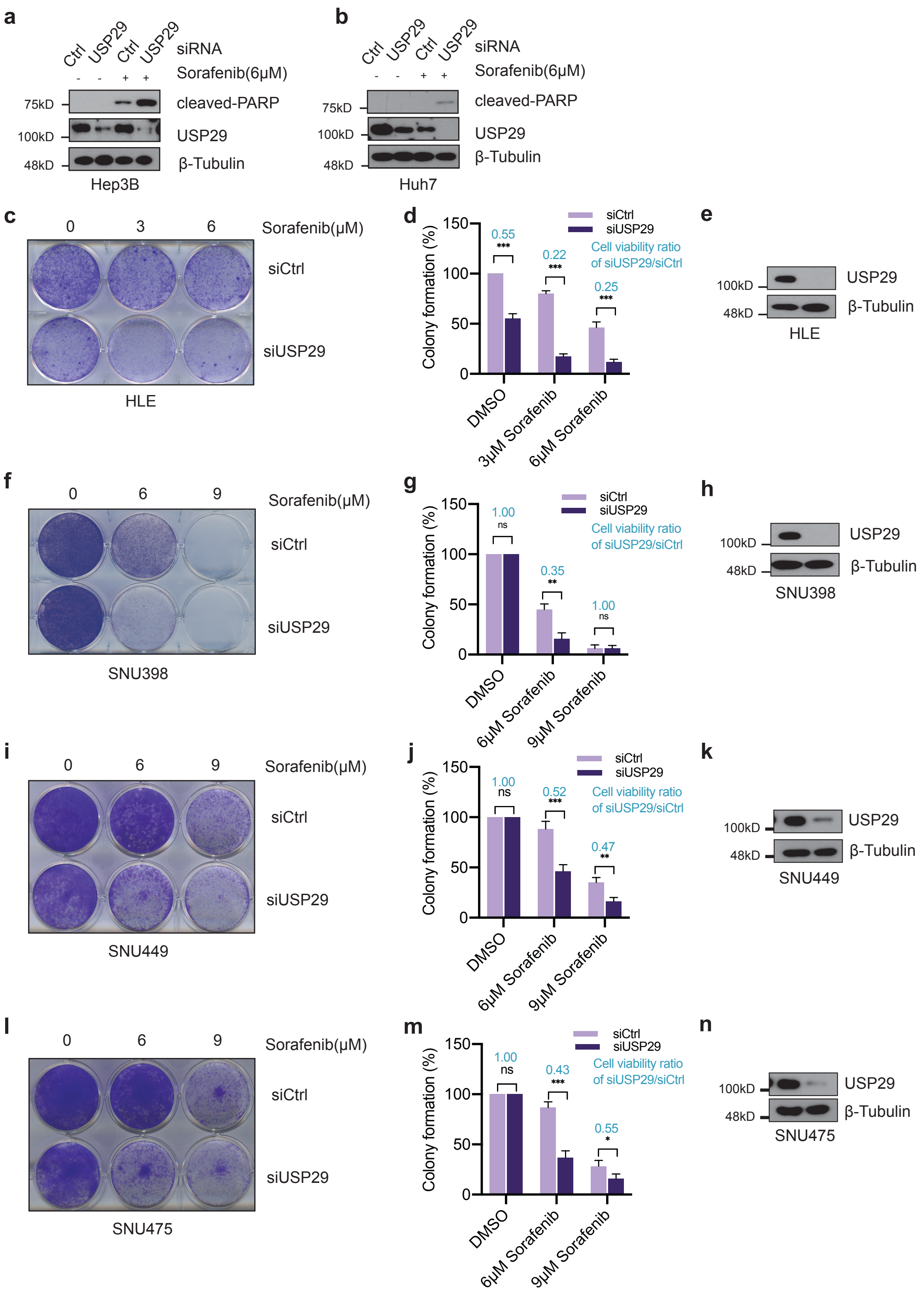

Suppl. Figure 4. USP29 deficiency promotes Sorafenib-induced cell death in intrinsic Sorafenib-resistant HCC cell lines.

(a,b) Loss of USP29 induces cell death upon Sorafenib treatment in Hep3B and Huh7 cells. Hep3B (a) and Huh7 (b) cells were transfected with siCtrl or ON-TARGET siUSP29 and treated with 6μM Sorafenib for 18 hours. Immunoblotting for cleaved PARP visualizes the levels of apoptosis. β-Tubulin was used as loading control. Results represent three independent experiments.

(c-n) USP29 deficiency diminishes cell survival in various Sorafenib-resistant HCC cell lines. Sorafenib-resistant cell lines HLE (c,d,e), SNU398 (f,g,h), SNU449 (i,j,k) and SNU475 (l,m,n) were transfected with siCtrl or ON-TARGET siUSP29 and plated for colony formation assays under treatment with different concentrations of Sorafenib (0μM, 3μM, 6μM or 0μM, 6μM, 9μM) for 2 weeks. Quantification of colony formation by crystal violet staining (d,g,j,m) revealed decreased cell survival upon loss of USP29 expression and Sorafenib treatment in comparison with controls. The ratio of cell viability between USP29-deficient and USP29-wildtype cells is given in blue numbers (d,g,j,m). n = 3 independent replicates. ns = not significant; *, $P < 0.05$; **, $P < 0.01$; ***, $P < 0.001$; Student's t-test. Knockdown efficiencies were validated by immunoblotting for USP29 (e,h,k,n). β-Tubulin was used as loading control. Results represent three independent experiments.

Suppl. Figure 5, Gao et al.

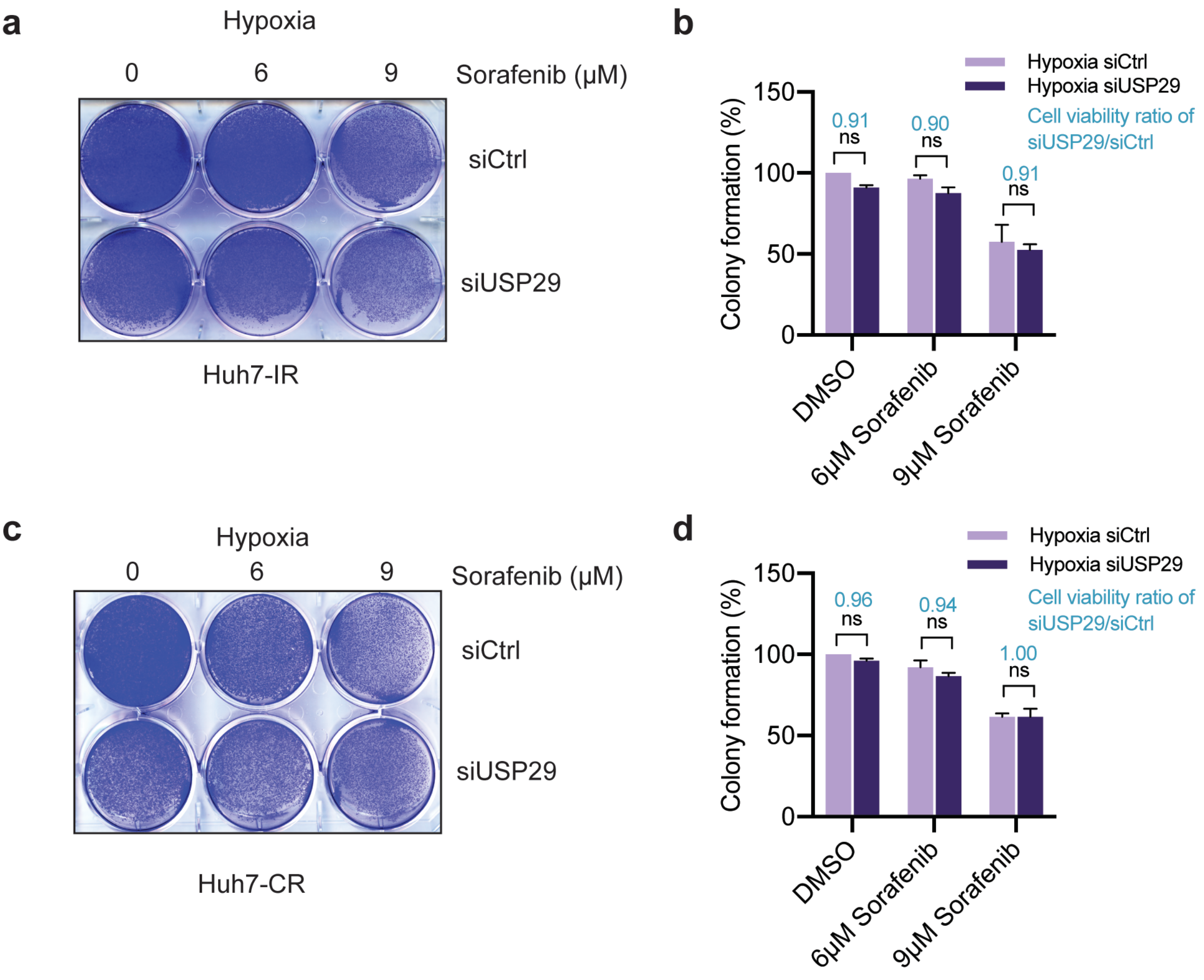

Suppl. Figure 5. Hypoxia rescues USP29 deficiency induced cell death in response to Sorafenib treatment in Sorafenib-resistant HCC cell lines.

(a-d) Hypoxia rescues USP29 deficiency-induced cell death in response to Sorafenib in Huh7-IR and Huh7-CR cells. Huh7-IR (a) and Huh-CR (b) cells were transfected with siCtrl or ON-TARGET siUSP29 every two days and plated for colony formation assays under treatment with different concentrations of Sorafenib (0μM, 6μM, 9μM) under hypoxia culture condition (1% O_2, 94% N_2, 5% CO_2) for 2 weeks, cell medium was refreshed every other day. Quantification of colony formation by crystal violet staining (b,d) revealed no decreased cell survival upon loss of USP29 expression and Sorafenib treatment in comparison with controls. The ratio of cell viability between USP29-deficient and USP29-wildtype cells is given in blue numbers (b,d). n = 3 independent replicates. ns = not significant; Student's t-test.

Suppl. Figure 6, Gao et al.

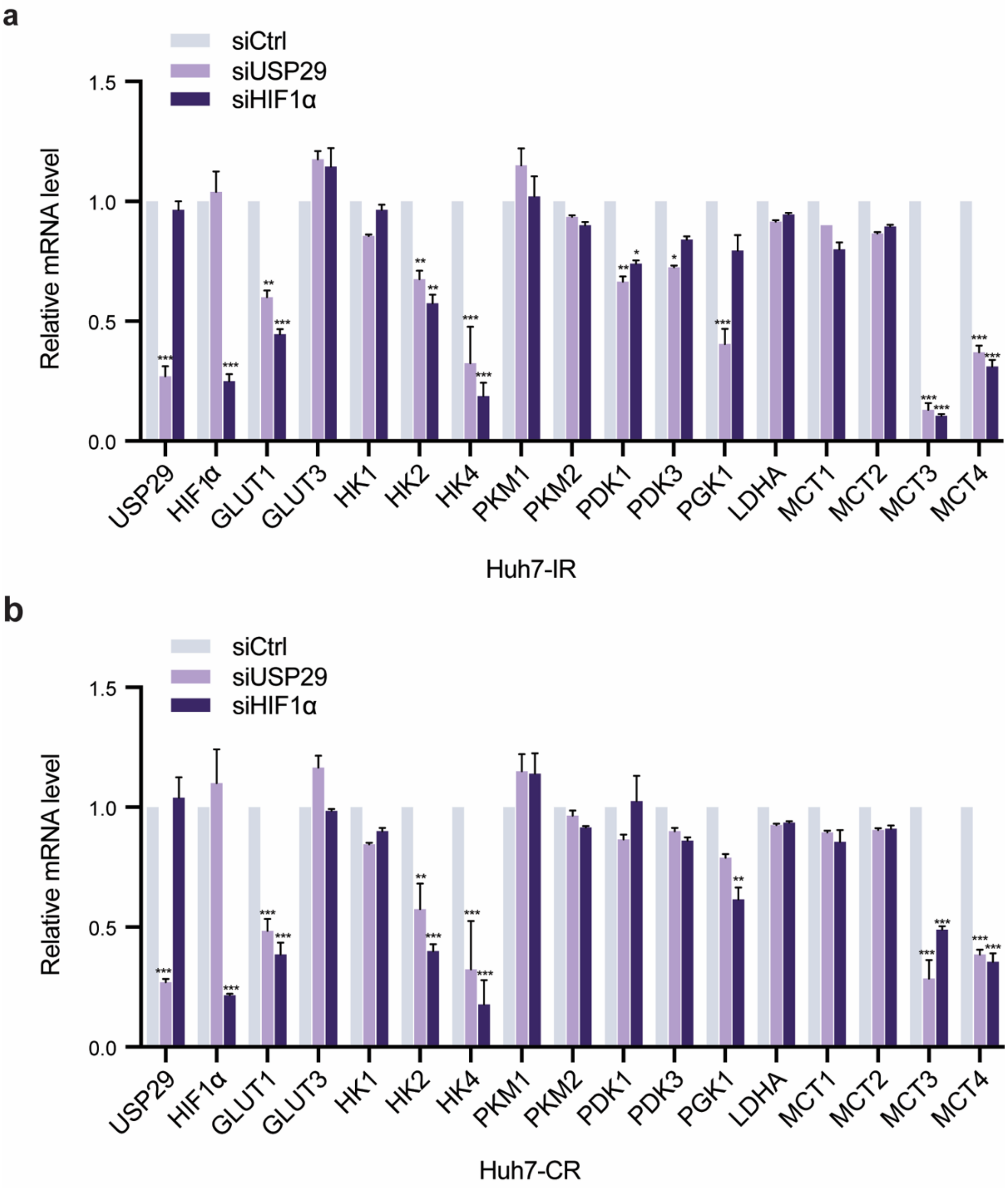

Suppl. Figure 6. The USP29-HIF1α axis regulates glycolysis in Sorafenib resistant cells. (a,b) Knockdown of USP29/HIF1α diminishes the expression of glycolysis-related genes in Sorafenib resistant Huh7-IR (a) and Huh7-CR (b) cells. Transcriptions of glycolysis metabolism were measured by quantitative RT-PCR with siRNA-mediated depletion of either USP29 or HIF1α. The expression *GLUT1, HK2*, *HK4*, *PDK1*, *PDK3*, *PGK1*, *MCT3* and *MCT4* showed decreased mRNA levels upon depletion of USP29 or HIF1α. n = 3 independent replicates). ns = not significant; *, $P < 0.05$; **, $P < 0.01$; ***, $P < 0.001$; Student's t-test.

Supplementary Tables

Suppl. Table I. Differential gene expression between Sorafenib-responsive and resistant cells

Excel file representing the differential gene expression between parental Sorafenib-sensitive Huh7 and Hep3B cells and their Sorafenib-resistant derivates Huh7-IR, Huh7-CR, Hep3B-IR and Hep3B-CR.

Suppl. Table II. siRNAs used in the study.

	SOURCE/Sequence (5' --- 3')
siCtrl	ON-TARGET plus non-targeting pool (D-001810-10-20)
siUSP8	ON-TARGET plus Human USP8 (L-005203-00-0005)
siUSP28	ON-TARGET plus Human USP28 (L-006076-00-0005)
siUSP29	ON-TARGET plus Human USP29 (L-006077-00-0005)
siUSP36	ON-TARGET plus Human USP36 (L-006084-00-0005)
siUSP37	ON-TARGET plus Human USP37 (L-006085-00-0005)
siUCHL1	ON-TARGET plus Human UCHL1 (L-004309-00-0005)
siHIF1α	ON-TARGET plus Human HIF1A (L-004018-00-0005)
siHIF2α	ON-TARGET plus Human EPAS1 (L-004814-00-0005)
siUSP29#1	CCCAUCAAGUUUAGAGGAUdTdT
siUSP29#2	GGAAUAUGCUGAAGGAAAUdTdT
shUSP29-Forward	GATCCCCCCATCAAGTTTAGAGGATTTCAAGAGAAT CCTCTAAACTTGATGGGTTTTTGGAAA
shUSP29-Reverse	AGCTTTTCCAAAAACCCATCAAGTTTAGAGGATTCTC TTGAAATCCTCTAAACTTGATGGGGG
Myc-USP29-RNAi-resistant-Forward	GGAAGAACCCTTCTAGCCTCGAAGACTTAGAAAAAGATA
Myc-USP29-RNAi-resistant-Reverse	TATCTTTTTCTAAGTCTTCGAGGCTAGAAGGGTTCTTCC

Suppl. Table III. Antibodies used in the study.

Antibodies	SOURCE	IDENTIFIER
HIF1α	Novus	NB100-449
HIF2α	Novus	NB100-122
USP29	Eurogentec	Peptide:1810359-1479
β-Tubulin	CST	2128
GLUT1	Abcam	ab652
Flag tag	Sigma	F1804
Myc tag	CST	2276
HA tag	CST	2362
Cleaved-PARP	CST	5625

Cleaved-caspase3	CST	9661
Ki67	Abcam	ab833

Suppl. Table IV. Oligonucleotides used in the study.

Oligonucleotides	5' --- 3'
hRPL19, forward primer	GATGCCGGAAAAACACCTTG
hPRL19, reverse primer	TGGCTGTACCCTTCCGCTT
HIF1α, forward primer	GAACGTCGAAAAGAAAAGTCTCG
HIF1α, reverse primer	CCTTATCAAGATGCGAACTCACA
USP29, forward primer	GGTGCCTCTTGACTCTCATTC
USP29, reverse primer	TGGGACACCTTGAGTGAGTAAG
GLUT1, forward primer	TTGCAGGCTTCTCCAACTGGAC
GLUT1, reverse primer	CAGAACCAGGAGCACAGTGAAG
GLUT3, forward primer	CAGAACCAGGAGCACAGTGAAG
GLUT3, reverse primer	TTGAACACCTGCATCCTTGA
HK1, forward primer	GCCACGATGAACCAAGGAATGG
HK1, reverse primer	GACAATG TGATCAAACAGCTC
HK2, reverse primer	GAGTTTGACCTGGATGTGGTTGC
HK2, forward primer	CCTCCATGTAGCAGGCATTGCT
HK4, reverse primer	CATCTCCGACTTCCTGGACAAG
HK4, forward primer	GAAGCTGGGGTGCAGCTTGTAC
PKM1, forward primer	CTATCCTCTGGAGGCTGTGC
PKM1, reverse primer	CGCACAAGTTCTTCAAACAGC
PKM2, forward primer	CTATCCTCTGGAGGCTGTGC
PKM2, reverse primer	GTGGGGTCGCTGGTAATG
LDHA, forward primer	GGATCTCCAACATGGCAGCCTT
LDHA, reverse primer	AGACGGCTTTCTCCCTCTTGCT
PDK1, forward primer	CATGTCACGCTGGGTAATGAGG
PDK1, reverse primer	CTCAACACGAGGTCTTGGTGCA
PDK3, forward primer	TGGAAGGAGTGGGTACTGATGC
PDK3, reverse primer	GGATTGCTCCAATCATCGGCTTC
MCT1, forward primer	TTGTTGGTGGCTGCTTGTCAGG
MCT1, reverse primer	TCATGGTCAGAGCTGGATTCAAG
MCT2, forward primer	TGCTGGCTGTTATGTACGCAGG
MCT2, reverse primer	TGCTGGCTGTTATGTACGCAGG
MCT3, forward primer	TGCAGTTCGAGGTGCTCATGGC
MCT3, reverse primer	GTTCTTCAACACATCCACCAGGC
MCT4, forward primer	CGTTCTGGGATGGGACTGAC
MCT4, reverse primer	ATGTGCCTCTGGACCATGTG
AXL, forward primer	GTTTGGAGCTGTGATGGAAGGC
AXL, reverse primer	CGCTTCACTCAGGAAATCCTCC
AHNAK2, forward primer	GAGAAGGAGGACACGGATGTTGC

AHNAK2, reverse primer	CCCCGCTTGCTCTTTATGGATTG
VEGFA, forward primer	AGGGCAGAATCATCACGAAGT
VEGFA, reverse primer	AGGGTCTCGATTGGATGGCA
USP8, forward primer	TTCCATTCAATACTTGGACCTGG
USP8, reverse primer	CCAAAGAGCCTTTAGCCAATGT
USP28, forward primer	CACTGTTGCTACAGAACCATCT
USP28, reverse primer	TGGGAGACTCCAGTAGACTCA
USP36, forward primer	TCTGCCAAGAAGGTCCTTTTACA
USP36, reverse primer	TGGCGACTAGCTCCCTCTG
USP37, forward primer	CCAGTGGAGCGAAACAAAGC
USP37, reverse primer	CCTCTGCATCCTTACTTGGTACT
UCHL1, forward primer	CCTGTGGCACAATCGGACTTA
UCHL1, reverse primer	CATCTACCCGACATTGGCCTT
